# Elevated FBXL6 activates both wild-type KRAS and mutant KRAS^G12D^ and drives HCC tumorigenesis via the ERK/mTOR/PRELID2/ROS axis in mice

**DOI:** 10.1186/s40779-023-00501-8

**Published:** 2023-12-20

**Authors:** Hao-Jun Xiong, Hong-Qiang Yu, Jie Zhang, Lei Fang, Di Wu, Xiao-Tong Lin, Chuan-Ming Xie

**Affiliations:** Key Laboratory of Hepatobiliary and Pancreatic Surgery, Institute of Hepatobiliary Surgery, Southwest Hospital, Army Medical University, Chongqing, 400038 China

**Keywords:** Ubiquitination, Kirsten rat sarcoma (KRAS), F-box and leucine-rich repeat 6 (FBXL6), PRELID2, Reactive oxygen species, Extracellular signal-regulated kinase (ERK), Mammalian target of rapamycin

## Abstract

**Background:**

Kirsten rat sarcoma (KRAS) and mutant KRAS^G12D^ have been implicated in human cancers, but it remains unclear whether their activation requires ubiquitination. This study aimed to investigate whether and how F-box and leucine-rich repeat 6 (FBXL6) regulates KRAS and KRAS^G12D^ activity in hepatocellular carcinoma (HCC).

**Methods:**

We constructed transgenic mouse strains LC (*LSL-Fbxl6*^*KI/*+^*;Alb-Cre*, *n* = 13), KC (*LSL-Kras*^*G12D/*+^*;Alb-Cre*, *n* = 10) and KLC (*LSL-Kras*^*G12D/*+^*;LSL-Fbxl6*^*KI/*+^*;Alb-Cre*, *n* = 12) mice, and then monitored HCC for 320 d. Multiomics approaches and pharmacological inhibitors were used to determine oncogenic signaling in the context of elevated FBXL6 and KRAS activation. Co‑immunoprecipitation (Co-IP), Western blotting, ubiquitination assay and RAS activity detection assay were employed to investigate the underlying molecular mechanism by which FBXL6 activates KRAS. The pathological relevance of the FBXL6/KRAS/extracellular signal-regulated kinase (ERK)/mammalian target of rapamycin (mTOR)/proteins of relevant evolutionary and lymphoid interest domain 2 (PRELID2) axis was evaluated in 129 paired samples from HCC patients.

**Results:**

FBXL6 is highly expressed in HCC as well as other human cancers (*P* < 0.001). Interestingly, FBXL6 drives HCC in transgenic mice. Mechanistically, elevated FBXL6 promotes the polyubiquitination of both wild-type KRAS and KRAS^G12D^ at lysine 128, leading to the activation of both KRAS and KRAS^G12D^ and promoting their binding to the serine/threonine-protein kinase RAF, which is followed by the activation of mitogen-activated protein kinase kinase (MEK)/ERK/mTOR signaling. The oncogenic activity of the MEK/ERK/mTOR axis relies on PRELID2, which induces reactive oxygen species (ROS) generation. Furthermore, hepatic FBXL6 upregulation facilitates KRAS^G12D^ to induce more severe hepatocarcinogenesis and lung metastasis via the MEK/ERK/mTOR/PRELID2/ROS axis. Dual inhibition of MEK and mTOR effectively suppresses tumor growth and metastasis in this subtype of cancer in vivo. In clinical samples, FBXL6 expression positively correlates with p-ERK (*χ*^2^ = 85.067, *P* < 0.001), p-mTOR (*χ*^2^ = 66.919, *P* < 0.001) and PRELID2 (*χ*^2^ = 20.891, *P* < 0.001). The Kaplan–Meier survival analyses suggested that HCC patients with high FBXL6/p-ERK levels predicted worse overall survival (log‑rank *P* < 0.001).

**Conclusions:**

FBXL6 activates KRAS or KRAS^G12D^ via ubiquitination at the site K128, leading to activation of the ERK/mTOR/PRELID2/ROS axis and tumorigenesis. Dual inhibition of MEK and mTOR effectively protects against FBXL6- and KRAS^G12D^-induced tumorigenesis, providing a potential therapeutic strategy to treat this aggressive subtype of liver cancer.

**Supplementary Information:**

The online version contains supplementary material available at 10.1186/s40779-023-00501-8.

## Background

Hepatocellular carcinoma (HCC), which usually develops in the context of chronic liver inflammation caused mainly by hepatitis B virus (HBV) or hepatitis C virus (HCV) infection, alcohol intake or metabolic syndrome, is the fourth leading cause of cancer-related mortality worldwide [1–4]. However, alternative therapeutics for advanced HCC are currently limited despite the enormous progress made in understanding the molecular biology of HCC. HCCs are heterogeneous and aggressive tumors with high molecular heterogeneity and unclear molecular targeting [5, 6], which has resulted in the lack of an effective systemic treatment that is suitable for all HCC patients with different genetic backgrounds. Therefore, it is imperative to explore potential novel methods to carefully stratify HCC patients and personalize therapeutic strategies.

The receptor tyrosine kinase (RTK)/rat sarcoma virus (RAS) signaling pathway is strongly activated in most human cancers, including HCC. Of all genetic alternations in HCC patients, genetic alteration of pathways related to RTK/RAS signaling is overrepresented, with 22–37% of HCC patients having at least one alteration in genes associated with the RTK/RAS/phosphatidylinositol 3‑kinase (PI3K) pathway [7, 8]. *RAS* or RAS signaling-related gene mutations that sustain RAS proteins in their GTP-binding forms occur in most human cancers. Unlike in other solid tumors (e.g., pancreatic tumors), *RAS* mutations are infrequent in HCC. In addition to oncogenic mutations, the overexpression of pan-Ras proteins that can also affect activity occurs in human cancers such as colorectal cancer (CRC) [9, 10] and a subset of breast cancers [11, 12]. Notably, mounting evidence has demonstrated that pan-Ras elevation also occurs in HCC, which leads to poor patient prognosis [13–15]. Specifically, Dietrich et al. [16] demonstrated that kirsten rat sarcoma (KRAS), an isoform of the RAS family (HRAS, KRAS, and NRAS), is dramatically overexpressed in HCC due to the loss of tumor-suppressive miRNA-622, activating the mitogen-activated protein kinase kinase (MEK)/extracellular signal-regulated kinase (ERK) and PI3K/serine/threonine kinase (Akt) signaling pathways and contributing to tumor progression, sorafenib sensitivity, and resistance. Recently, a research group found that WD repeat domain 76 (WDR76) promotes the polyubiquitination-dependent degradation of RAS, which results in the inhibition of proliferation, transformation, and invasion of liver cancer cells [17]. However, whether KRAS can be activated by ubiquitination in HCC has not yet been reported.

F-box and leucine-rich repeat 6 (FBXL6), a member of the F-box family, was first reported to degrade ETS variant transcription factor 6 (ETV6) via the ubiquitin–proteasome system and, as a result, to participate in cell development and differentiation [18]. One recent study demonstrated that FBXL6 targets phospho-p53 (S315) for polyubiquitination and proteasomal degradation, thereby inhibiting p53 signaling, which leads to the proliferation of CRC cells [19]. This result revealed the tumor-promoting effects of FBXL6. Another study showed that FBXL6 promotes the K63-linked ubiquitination of heat shock protein 90 alpha family class A member 1 (HSP90AA1), contributing to the stabilization and activation of c-Myc and leading to the growth of HCC cells [20]. Although FBXL6 has been proven to have a tumor-promoting effect in several cancers, the mechanisms by which FBXL6 promotes HCC remain unclear, especially in vivo.

In this study, we aimed to investigate whether and how FBXL6 regulates KRAS/KRAS^G12D^ ubiquitination and activity. Here, we report that FBXL6 activates KRAS/KRAS^G12D^ by enhancing their ubiquitination at the site K128, leading to activation of the MEK/ERK/mammalian target of rapamycin (mTOR) axis, which further facilitates KRAS^G12D^-induced hepatocarcinogenesis and lung metastasis in transgenic mice. Dual inhibition of MEK and mTOR effectively suppressed KRAS mutant- and FBXL6-driven HCC, and this finding may provide a potential treatment strategy for this cohort of HCC patients with high FBXL6 expression and high RAS/ERK activation.

## Methods

### Mouse strain generation and mouse breeding

Mice containing Loxp-STOP-Loxp-Fbxl6 (*LSL-Fbxl6*^*KI/*+^, *n* = 5) were generated by Biocytogen Pharmaceuticals (Beijing, China), and floxed alleles of Fbxl6 (conditional knockout mice of Fbxl6, *CKO-Fbxl6*^*KO/*+^, *n* = 4) were generated by Cyagen Biosciences Corporation (Guangzhou, China). Loxp-STOP-Loxp-Kras^G12D^ (*LSL-Kras*^*G12D/*+^) and liver-specific Cre recombinase strain *Alb-Cre* mice were purchased from The Jackson Laboratory (Bar Harbor, ME, USA). *LSL-Fbxl6*^*KI/*+^ and *CKO-Fbxl6*^*KO/*+^ mice were crossed with *Alb-Cre* mice to generate *LSL-Fbxl6*^*KI/*+^*;Alb-Cre* (hereinafter referred to as LC mice,* n* = 13) and *CKO-Fbxl6*^*KO/*+^*;Alb-Cre* mice (*n* = 6). In addition, *LSL-Kras*^*G12D/*+^*;Alb-Cre* mice (hereinafter referred to as KC mice, *n* = 10) were obtained by crossing *LSL-Kras*^*G12D/*+^ mice with *Alb-Cre* mice. The LC mice were crossed with KC mice to generate *LSL-Kras*^*G12D/*+^*;LSL-Fbxl6*^*KI/*+^*;Alb-Cre* (hereinafter referred to as KLC, *n* = 12) mice. *CKO-Fbxl6*^*KO/*+^*;Alb-Cre* mice were crossed with KC mice to generate *LSL-Kras*^*G12D/*+^*;CKO-Fbxl6*^*KO/*+^*;Alb-Cre* mice (*n* = 5)*.* The abovementioned KC, LC and KLC mouse strains were genotyped by PCR amplification methods (Additional file 1: Fig. S1a–d). The corresponding primers for genotyping are listed in Additional file 1: Table S1. All mouse-related experiments and operations were approved by the Institutional Animal Care and Use Committees of Army Medical University (No. AMUWEC20211905) and met the standards of the Office of Laboratory Animal Welfare and Chinese Animal Protection Laws.

### Ubiquitome analysis

The LC and *Alb-Cre* mice were raised in the SPF mouse facility for 320 d, after which the mice were anesthetized with isoflurane and their livers (containing the spontaneous tumors) dissected, followed by ubiquitome analysis.

### Chemicals and antibodies

Cycloheximide (CHX; S7418) and MG132 (S2619) were obtained from Selleck Corporation (Houston, TX, USA). Nickel-nitrilotriacetic acid (Ni–NTA) agarose beads (30210) were purchased from Qiagen Company (Duesseldorf, Germany). Everolimus (HY-10218) and trametinib (HY-10999) were obtained from MedChemExpress (MCE) Corporation (Monmouth Junction, NJ, USA). Anti-p-mTOR (5536), anti-mTOR (2983), anti-p-Akt (4060), anti-Akt (4691), anti-p-ERK (4376), anti-ERK (4695), anti-p-S6 (2211), anti-S6 (2217), anti-p-70S6K (9234), anti-70S6K (9202), anti-p-eukaryotic translation initiation factor 4E binding protein 1 (4EBP1) (2855), and anti-4EBP1 (9644) antibodies were purchased from Cell Signaling Technology (Danvers, MA, USA). Anti-proteins of relevant evolutionary and lymphoid interest (PRELI) domain 2 (PRELID2) antibody (NBP1-81937) was obtained from Novus Biologicals (Littleton, CO, USA). Anti-KRAS antibody (ab275876) was purchased from Abcam (Cambridge, MA, USA). Anti-β-actin (20536-1-AP) and anti-GAPDH (10494-1-AP) antibodies were purchased from Proteintech Company (Wuhan, China). Anti-hemagglutinin (HA) antibody (11867423001) was purchased from Roche (Basel, Switzerland). Anti-Flag antibody (F1804) was purchased from Sigma-Aldrich (St. Louis, MO, USA). Goat anti-mouse IgG horseradish peroxidase (HRP), goat anti-rabbit IgG HRP and goat anti-rat IgG HRP were purchased from Cell Signaling Technology (Danvers, MA, USA).

### Plasmid construction

The pcDNA3.1-hUb-his, pcDNA3.1-hFBXL6-Flag, pcDNA3.1-hKRAS-HA, and pcDNA3.1-mPrelid2-Flag plasmids were constructed by Genechem Corporation (Shanghai, China). KRAS^G12D^ (pcDNA3.1-hKRASs^G12D^-HA), KRAS^K128R^ (pcDNA3.1-hKRAS^K128R^-HA), KRAS^K117R^ (pcDNA3.1-hKRAS^K117R^-HA), KRAS^K147R^ (pcDNA3.1-hKRAS^K147R^-HA), KRAS^K170R^ (pcDNA3.1-hKRAS^K170R^-HA), and KRAS containing the double mutations G12D and K128R (pcDNA3.1-hKRAS^DM^-HA) were generated by PCR amplification using the QuikChange Lightning Site-Directed Mutagenesis Kit (Agilent, USA) based on the pcDNA3.1-hKRAS-HA plasmid. The primers used for site-directed mutagenesis were designed with an online tool supported by Agilent Corporation and are listed in Additional file 1: Table S2.

### Cell culture

The Huh7 cell line was obtained from the Japanese Collection of Research Bioresources and authenticated by short tandem repeat (STR) profiling. The Hep3B cell line and HEK293T cell line were purchased from the American Type Culture Collection (ATCC). Huh7, Hep3B and HEK293T cells were cultured in Dulbecco’s modified Eagle’s medium (DMEM) supplemented with 10% fetal bovine serum (FBS) in a 37 °C incubator with 5% CO_2_.

### Transient transfection

After seeding and growth to 80% confluence in a culture plate, the cells were transfected with plasmids or siRNAs using Lipofectamine 2000 from Invitrogen (Carlsbad, CA, USA) according to the manufacturer’s instructions. siRNAs targeting Prelid2 were designed and synthesized by GenePharma (Shanghai, China), and the siRNA sequences are listed in Additional file 1: Table S3.

### Co-immunoprecipitation (Co-IP)

After treatment with the corresponding methods, the cells were washed with ice-cold PBS and then lysed in lysis buffer (Beyotime, Shanghai, China) supplemented with protease inhibitor (Roche, Basel, Switzerland) for 20 min. After centrifugation at 13,000 r/min for 10 min at 4 °C, the cell lysates were precleared with protein A/G agarose beads (Santa Cruz, CA, USA). Thereafter, rabbit or mouse IgG negative control (CST, Danvers, MA, USA) or the corresponding primary antibodies were added to the lysates at a ratio of 2 μg per 1 mg of total protein and then incubated for 1 h at 4 °C. Subsequently, 50 µl of protein A/G PLUS-agarose (Santa Cruz, CA, USA) was added and incubated on a rotating device overnight at 4 °C. Afterward, the mixtures were washed using ice-cold PBS 3 times and boiled with 1 × SDS-PAGE loading buffer, and then the supernatants were utilized for Western blotting.

### Western blotting

Cells or tissues were lysed with RIPA lysis buffer (Beyotime, Shanghai, China) containing a protease inhibitor mixture (Roche, Switzerland) and then centrifuged at 13,000 rpm for 15 min at 4 °C. Subsequently, the supernatants were transferred to new Eppendorf tubes, and then the protein concentrations were determined using a BCA Protein Assay Kit (Beyotime, Shanghai, China). Thereafter, the normalized protein solutions were denatured and separated by SDS-PAGE (Beyotime, Shanghai, China), after which the proteins were transferred to NC membranes (GE Healthcare, UK). After blocking with 5% skim milk, the NC membranes were incubated with the indicated primary antibodies at 4 °C overnight. After incubation with HRP-conjugated secondary antibodies for 1 h at 25 °C, the specific protein signal was detected using chemiluminescence detection reagents (Millipore Corporation, Temecula, CA, USA) and a ChemiDoc imaging system (Bio-Rad, Hercules, CA, USA).

### In vivo ubiquitination assay

An in vivo ubiquitination assay was performed as follows. HEK293T cells were cotransfected with HA-KRAS and His-Ub (or the indicated His-Ub mutant) plasmids in the presence or absence of Flag-FBXL6 for 72 h and then exposed to MG132 (10 μmol/L) for 4 h before harvest. Subsequently, the cells were harvested for the in vivo ubiquitination assay. First, after washing two times with ice-cold PBS, the cells were lysed with buffer A [6 mol/L guanidinium-HCl, 0.1 mol/L Na_2_HPO_4_/NaH_2_PO_4_, 10 mmol/L Tris–HCl (pH 8.0), 5 mmol/L imidazole, and 10 mmol/L β-mercaptoethanol] on ice for 20 min, followed by sonication to reduce viscosity. Thereafter, 50 μl of Ni–NTA-agarose beads (Qiagen, Valencia, CA) was added to the cleared cell lysates and rotated gently overnight at 4 °C. Second, the beads were successively washed with buffer A plus 10 mmol/L β-mercaptoethanol, buffer B [8 mmol/L urea, 0.1 mol/L Na_2_HPO_4_/NaH_2_PO_4_, 10 mmol/L Tris/HCl (pH 8.0), and 10 mmol/L β-mercaptoethanol], buffer C [8 mmol/L urea, 0.1 mol/L Na_2_HPO_4_/NaH_2_PO_4_, 10 mmol/L Tris/HCl (pH 6.3), 10 mmol/L β-mercaptoethanol, and 0.2% Triton X-100], and buffer C plus 10 mmol/L β-mercaptoethanol and 0.1% Triton X-100. Finally, the beads were eluted with buffer D [200 mmol/L imidazole, 0.15 mol/L Tris–HCl (pH 6.7), 30% glycerol, 0.72 mol/L β-mercaptoethanol, and 5% SDS] and then detected using Western blotting.

### RAS activity detection

RAS activity was determined using the RAS Activation Assay Kit (NewEast Bioscience, King of Prussia, PA, USA) according to the manufacturer’s instructions. Briefly, after washing with ice-cold PBS two times, the cells were lysed with ice-cold 1 × assay/lysis buffer on ice for 15 min. Subsequently, the cell lysates were centrifuged (11,200 r/min) for 10 min at 4 °C, and then the clear supernatant was transferred to another Eppendorf tube, followed by mixing with anti-active RAS monoclonal antibody and protein A/G agarose beads, and then incubated in tubes at 4 °C for 1 h with gentle agitation. Thereafter, the beads were washed three times with 0.5 ml of 1 × assay/lysis buffer, denatured with 2 × reducing SDS-PAGE sample buffer and detected by Western blotting.

### RNA extraction and quantitative PCR (qPCR)

Total RNA was extracted from HCC cell lines and liver tissues using RNAiso Plus reagent (TaKaRa, Japan), and cDNA was synthesized using the PrimeScript RT Reagent Kit (TaKaRa, Japan) according to the manufacturer’s instructions. The CFX96 Touch Real-time PCR Detection System from Bio-Rad Laboratories (Hercules, CA, USA) and Premix Ex Taq II (TaKaRa, Japan) were utilized to perform qPCR according to the manufacturers’ protocols. The relative fold changes in target genes were determined using the 2^−ΔΔCt^ method, and β-actin was used as an internal control. The sequences of the primers used for qPCR analysis are listed in Additional file 1: Table S4.

### Primary cell isolation

The isolation of primary cells from the liver was performed as follows. Briefly, the residual blood cells in the liver were washed away with an EGTA solution perfused through the inferior vena cava (IVC) for 2 min, followed by perfusion with a pronase solution (0.4 mg/ml) and collagenase solution (0.8 mg/ml) for 5 and 7 min, respectively. Thereafter, the liver was dissected and digested with 5 ml of a pronase/collagenase solution (containing 2 mg of pronase, 2.5 mg of collagenase and 1% DNase I) for 25 min. The resulting solution (mixed with small tissue cubes) was filtered through a 70-µm nylon mesh, followed by centrifugation at 4 °C and washing with Gey’s balanced salt solution (GBSS). Primary cells were successfully obtained. These cells were cultured in a 37 °C incubator with DMEM supplemented with 20% FBS and 2% penicillin/streptomycin.

### Immunohistochemistry (IHC) and HE staining

Dissected liver tissues were fixed using 10% paraformaldehyde overnight and then embedded in paraffin, followed by consecutive slicing to a thickness of 3–5 mm. For the IHC assay, the rehydrated tissues were treated with citrate buffer (pH 6.0; 10 mmol/L citric acid, 0.05% Tween 20) and then heated in a high-pressure boiler for antigen retrieval. Thereafter, the slides were incubated with methanol containing 3% hydrogen peroxide for 15 min to quench endogenous peroxidase activity, followed by blocking with TBS containing 3% goat serum. Subsequently, the sections were incubated with the indicated primary antibodies at 4 °C overnight, incubated with the corresponding secondary antibody at room temperature for 60 min, stained with diaminobenzidine and counterstained with hematoxylin. For HE staining, the tissue sections were successively dewaxed, rehydrated (in a graded ethanol series) and stained with hematoxylin using routine methods and then stained with eosin for approximately 20 s. Afterward, the tissues were dehydrated and covered with neutral gum. A light microscope was utilized to collect images.

### Cell counting kit-8 (CCK-8) assay

A CCK-8 kit (Dojindo, Kumamoto, Japan) was used to measure cell viability. Briefly, HCC cells (approximately 3000 cells per well) were seeded in a 96-well plate overnight and then transfected with plasmids (or siRNAs) or treated with the respective reagents for the indicated times. Thereafter, CCK-8 agents were added to each well at a ratio of 10 μl per well and then incubated at 37 °C for 30–60 min, followed by detection of the absorption value at 450 nm with a microplate reader (Thermo Scientific, Waltham, MA, USA).

### Transcriptome profiling

The LC, KC and KLC mice were raised in a specific-pathogen-free (SPF) mouse facility for approximately 30 weeks and anesthetized with isoflurane, and the livers (containing spontaneous tumors) were dissected. Subsequently, the tumors from the above three groups (each group contained three individuals) were obtained and sent for transcriptome profiling analysis (Genergy Bio, Shanghai, China). The original transcriptome profiling data will be available in a public database when needed.

### Transwell assay

KLC primary cells were seeded in a 6-well plate overnight and then transfected with the indicated siRNAs using Lipofectamine 2000 according to the manufacturer’s protocol for 48 h. Thereafter, the cells (approximately 5 × 10^4^) were digested with trypsin, suspended in 100 µl of serum-free DMEM, and seeded in the top chamber of a Transwell (8 μm, 24-well format, Millipore, Billerica, MA, USA). In addition, complete medium with serum (500 µl) containing 20% FBS was added to the bottom chamber. After a 24 h incubation period, the chambers were fixed with 4% paraformaldehyde for 30 min and stained with crystal violet (Beyotime, Shanghai, China). Photographs of three randomly selected fields of the fixed cells were captured, and the cells in the imaged fields were counted. Three independent replicates of each experiment were performed.

### Reactive oxygen species (ROS) assay

KLC primary cells (3 × 10^5^) were seeded in 6-well plates overnight and then exposed to different treatments for the indicated times, followed by ROS detection. Briefly, the treated cells were washed two times with PBS and then incubated with the fluorescent probe 2′,7′-dichlorodihydrofluorescein diacetate (DCFH-DA) (S0033, Beyotime, Shanghai, China) for 25 min. Subsequently, the fluorescence intensity in the cells was detected using a fluorescence microscope.

### Construction of KLC primary cells with stable *Prelid2* knockdown

Packaged lentiviruses containing sh-Prelid2 (shRNA-Prelid2) or negative control (sh-NC) were designed and synthesized by Genechem Corporation (Shanghai, China). These lentiviruses were used to infect KLC primary cells at a multiplicity of infection (MOI) of 10, followed by screening with puromycin, and the resulting KLC primary cells with stable *Prelid2* knockdown were named LV-shPrelid2-1, LV-shPrelid2-2 and LV-shPrelid2-3. The negative control KLC primary cells with stable expression of si-NC were named LV-shNC. The sequences of the shRNAs are listed in Additional file 1: Table S5.

### Xenograft tumor assay in nude mice

Six-week-old male mice were purchased from Beijing Huafukang Bioscience (Beijing, China) and raised in the laboratory animal center of Army Medical University (Chongqing, China). The mice were randomly divided into two groups, and one group was subcutaneously injected with KLC primary cells with stable *Prelid2* knockdown in the axillae. The other group was subcutaneously injected with LV-shNC KLC primary cells. The tumor size was measured with calipers every other day, and the tumor volume was calculated using the following formula: V = length × width^2^ × 1/2. The tumor growth curves were plotted using tumor volume at the indicated time points. The tumor volumes were recorded for 15 d before the mice were euthanized. All animal experiments were performed in accordance with the guidelines of the Laboratory Animal Center at the Army Medical University and approved by the Animal Ethics Committee.

### Orthotopic HCC tumor model

Six- to eight-week-old BALB/c male nude mice were obtained and raised in the animal center of Southwest Hospital of Army Medical University (Chongqing, China), and then KLC primary cells were subcutaneously injected into the right axillae of the nude mice. Once the tumor volume reached approximately 150 mm^3^, the mice were euthanized. After washing with normal saline, the freshly dissected tumors were cut into small tissue blocks (approximately 3–5 mm) and then orthotopically implanted into the mouse livers. Seven days later, the mice were randomized into three groups: a negative control (NC) group, an everolimus (E) group and a combined everolimus and trametinib (E + T) group. The NC group was treated with vehicle, the E group was treated with everolimus (diluted in DMSO and coil oil, 4 mg/kg, intraperitoneal injection) twice per week for six rounds, and the E + T group was treated with both everolimus (everolimus was dosed as in the E group) and trametinib (diluted in DMSO and coil oil, 0.5 mg/kg, intraperitoneal injection) twice per week for six rounds. Orthotopic tumors from different groups were obtained 5 d after the last injection. The tumor size was measured with calipers, and the tumor volume was calculated using the following formula: V = length × width^2^ × 1/2. Finally, the dissected tumors were divided into several parts for HE and IHC staining.

### HCC patient tumor samples

One hundred and twenty-nine HCC patients who underwent HCC resection surgery at Southwest Hospital of Army Medical University between January 2012 and December 2015 were included in this study with the approval of the Ethics Committee of Southwest Hospital of Army Medical University (No. KY2020127). Corresponding data (including age, gender, TNM stage, histologic grade, tumor size, recurrence, vascular thrombosis, metastasis and outcome) of these HCC patients were obtained from the Clinical Research Center of the Institution of Hepatobiliary Surgery at Southwest Hospital of Army Medical University. The following were key inclusion criteria: HCC patients with primary HCC only (cholangiocellular carcinoma, or mixed liver cancer, was excluded), 18–70 years of age, no therapy for liver cancer before surgery, and HCC radical surgery (R0) by open or minimally invasive surgery. The key exclusion criteria were as follows: (1) primary HCC patients who died from other causes, (2) primary HCC patients with other primary cancers, and (3) patients with primary HCC who were unwilling to attend appointments for this research. Univariate or multivariate survival analysis was carried out using the Cox proportional hazards model. In addition, the clinical and pathological data of 365 HCC patients were downloaded from The Cancer Genome Atlas (TCGA) database.

### Statistical analysis

GraphPad Prism 8.0 software (San Diego, CA, USA) or IBM SPSS 24.0 software (IBM, New York, NY, USA) was used for statistical analysis. Unpaired Student’s* t*-test was used for comparisons of two groups. Comparisons between more than two groups were performed using ANOVA with Tukey’s or Bonferroni’s multiple comparisons test. The Pearson *χ*^*2*^ test or Fisher’s exact test was utilized to compare the distribution of categorical factors between the different groups. The Kaplan–Meier method was utilized to perform survival analysis, which was carried out by the log-rank test. The measurement data are shown as the mean ± standard error of the mean (SEM) from at least three independent experiments, while the counting data are described in the form of frequency or percentage. Differences for which *P* < 0.05 were considered statistically significant.

## Results

### FBXL6 activates KRAS and KRAS^G12D^ by K63-linked polyubiquitination at the site K128

**Fig. 1:**
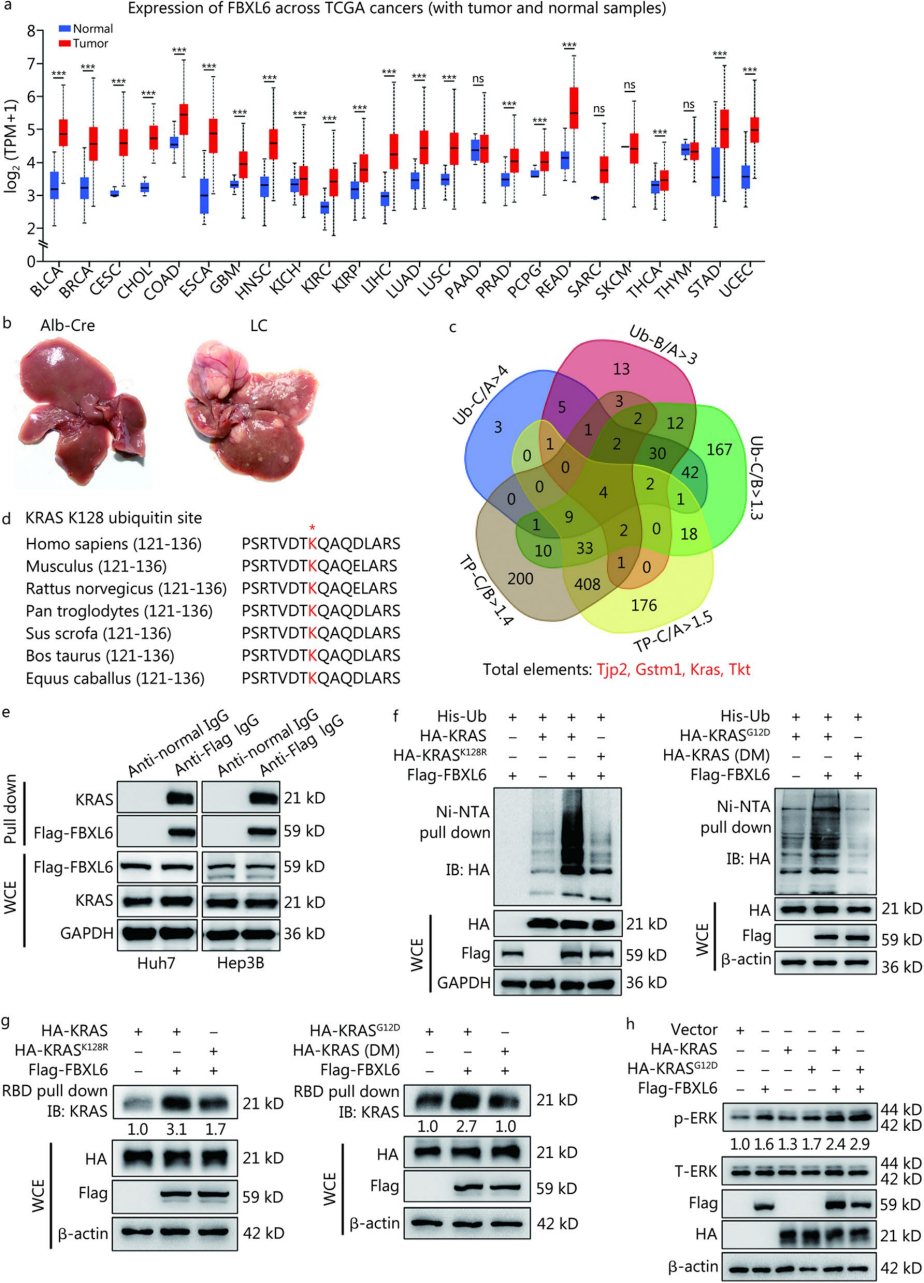
FBXL6 activates KRAS and KRAS^G12D^ by K63-linked polyubiquitination at the site K128. **a** Analysis of FBXL6 expression across cancers in the TCGA database shows that FBXL6 is upregulated in various types of human cancers, including liver hepatocellular carcinoma (LIHC), namely, HCC. **b** *Alb-Cre and LSL-Fbxl6*^*KI/*+^*;Alb-Cre* (LC) male mice were monitored for 320 d and then euthanized. The livers were imaged. **c** Diagram showing the number of proteins with increased ubiquitinated sites and upregulated expression in HCC tumors. Ub-C/A > 4 indicates proteins with a more than fourfold increase in ubiquitinated sites in tumors compared with normal tissues from male mice; Ub-B/A > 3 indicates proteins with a more than threefold increase in ubiquitinated sites in adjacent tissues compared with normal tissues. Ub-C/B > 1.3 indicates proteins with a more than 1.3-fold increase in ubiquitinated sites in tumors compared with adjacent tissues. TP-C/B > 1.4 and TP-C/A > 1.5 indicate that the protein levels were upregulated 1.4- or 1.5-fold in tumors compared with adjacent tissues or normal tissues, respectively. **d** Evolutionary conservation of the site K128 on KRAS from different species. A red star (*) indicates a conserved site in KRAS in different species. **e** A co-IP assay was utilized to measure the interaction between FBXL6 and KRAS. Huh7 and Hep3B cells were transfected with Flag-FBXL6 plasmids for 48 h and then lysed. The indicated antibodies and protein A/G PLUS-Agarose were added to the cell lysates. **f** HEK293T cells were transfected with the indicated plasmids for 72 h and then lysed with a 6 mol/L guanidine solution, followed by pull-down using Ni–NTA beads or direct Western blotting with the indicated antibodies. **g** Huh7 cells were transfected with the indicated plasmids for 72 h and lysed with lysis buffer. Activated KRAS was pulled down with an anti-active RAS monoclonal antibody (RBD peptide), followed by Western blotting. Band intensity was quantified by ImageJ software. **h** After serum deprivation for 12 h, Huh7 cells were transfected with the indicated plasmids for 72 h, followed by Western blotting. ns non‑significant; ^***^P < 0.001. BLCA bladder urothelial carcinoma, BRCA breast invasive carcinoma, CESC cervical squamous cell carcinoma and endocervical adenocarcinoma, CHOL cholangiocarcinoma, COAD colon adenocarcinoma, ESCA esophageal carcinoma, GBM glioblastoma multiforme, HNSC head and neck squamous cell carcinoma, KICH kidney chromophobe, KIRC kidney renal clear cell carcinoma, KIRP kidney renal papillary cell carcinoma, LUAD lung adenocarcinoma, LUSC lung squamous cell carcinoma, PAAD pancreatic adenocarcinoma, PRAD prostate adenocarcinoma, PCPG pheochromocytoma and paraganglioma, READ rectum adenocarcinoma, SARC sarcoma, SKCM skin cutaneous melanoma, THCA thyroid carcinoma, THYM thymoma, STAD stomach adenocarcinoma, UCEC uterine corpus endometrial carcinoma, FBXL6 F-box and leucine-rich repeat 6, TCGA The Cancer Genome Atlas, WCE whole-cell extract, HA hemagglutinin, KRAS kirsten rat sarcoma, KRAS^G12D^ glycine to aspartic acid mutation of KRAS, Ub ubiquitin, HA-KRAS (DM) double mutation of KRAS at the sites K128 and G12, A normal liver tissue, B adjacent tumor tissue, C cancer or tumor tissue, TP total protein, K128 lysine 128, Co-IP coimmunoprecipitation, RBD RAS-binding domain

The TCGA database showed that FBXL6, an Skp1-Cullin-F box (SCF) E3 ligase, is highly expressed in most human cancers, including liver hepatocellular carcinoma (LIHC), namely, HCC (*P* < 0.001, Fig. 1a). Here, we established a transgenic mouse model with high liver-specific expression of Fbxl6, named *LSL-Fbxl6*^*KI/*+^*;Alb-Cre* (LC). As shown in Additional file 1: Fig. S1e-f, mRNA and protein expression of *Fbxl6* in the liver was dramatically upregulated (approximately 40-fold) in the LC mice compared with the WT mice (*P* < 0.01), and this finding was further validated by IHC staining (Additional file 1: Fig. S1g). After monitoring LC mice for 320 d, we found that LC mice spontaneously developed HCC tumors (Fig. 1b). Compared with that in normal tissues from *Alb-Cre* mice (liver-specific Cre recombinase line), the ubiquitination of KRAS at the site lysine 128 (K128) was increased nearly 4.4-fold in LC tumor tissues (Fig. 1c), suggesting that KRAS ubiquitination may be involved in Fbxl6-mediated hepatocarcinogenesis. Furthermore, we found that the site K128 in KRAS was highly conserved across species (Fig. 1d). Therefore, we next determined whether KRAS was a direct substrate of FBXL6. As shown in Additional file 1: Fig. S2a, exogenously expressed HA-KRAS protein could be immunoprecipitated by anti-Flag antibody targeting ectopically expressed Flag-FBXL6. Moreover, ectopically expressed FBXL6 pulled down endogenous KRAS from Huh7 and Hep3B cells (Fig. 1e). These results indicated that FBXL6 could interact with KRAS. We further explored whether the site K128 in KRAS/KRAS^G12D^ was ubiquitylated by FBXL6. Therefore, we separately generated two mutant plasmids named HA-KRAS^K128R^ (lysine mutated to arginine at the site K128 in KRAS) and HA-KRAS (DM) (double mutation of KRAS, namely, glycine mutated to aspartic acid at the site G12 in HA-KRAS^K128R^). The results showed that overexpression of FBXL6 dramatically enhanced the polyubiquitination of KRAS and its mutant (KRAS^G12D^), which was remarkably inhibited by the mutation K128R (Fig. 1f), indicating that FBXL6 promoted the polyubiquitination of KRAS and its G12D mutant at the site K128. Considering that three other lysine sites (K117, K147 and K170) have been identified as the ubiquitination sites of RAS [21–23], we explored whether these lysine sites could be influenced by FBXL6. The results showed that none of the corresponding mutants (K117R, K147R and K170R) influenced FBXL6-mediated KRAS ubiquitination (Additional file 1: Fig. S2b), which strengthens the importance of K128 polyubiquitination in FBXL6-mediated KRAS ubiquitination.

Generally, K63-linked polyubiquitination of a target protein leads to its activation, whereas K48-linked polyubiquitination induces proteasomal degradation [24]. Therefore, we determined the type of ubiquitin chain linkage in KRAS that was induced by FBXL6. The results showed that FBXL6 promoted the K63-linked polyubiquitylation of KRAS (Additional file 1: Fig. S2c, d), suggesting that FBXL6 may activate KRAS. We subsequently explored whether FBXL6 influenced the activity of KRAS or KRAS^G12D^ and found that FBXL6 enhanced the activity of KRAS and KRAS^G12D^, whereas the mutant K128R attenuated this action, as indicated by the active KRAS-RBD interface (Fig. 1g). To further validate the activation of KRAS by FBXL6, we detected the downstream effector of KRAS (namely, ERK) and found that FBXL6 further enhanced KRAS- or KRAS^G12D^-induced ERK activation under starvation or normal conditions (Fig. 1h, Additional file 1: Fig. S2e). Collectively, these results indicated that FBXL6 activates both KRAS and KRAS^G12D^ by K63-linked polyubiquitination at the site K128.

### FBXL6 facilitates KRAS^G12D^-driven hepatocarcinogenesis and lung metastasis

**Fig. 2:**
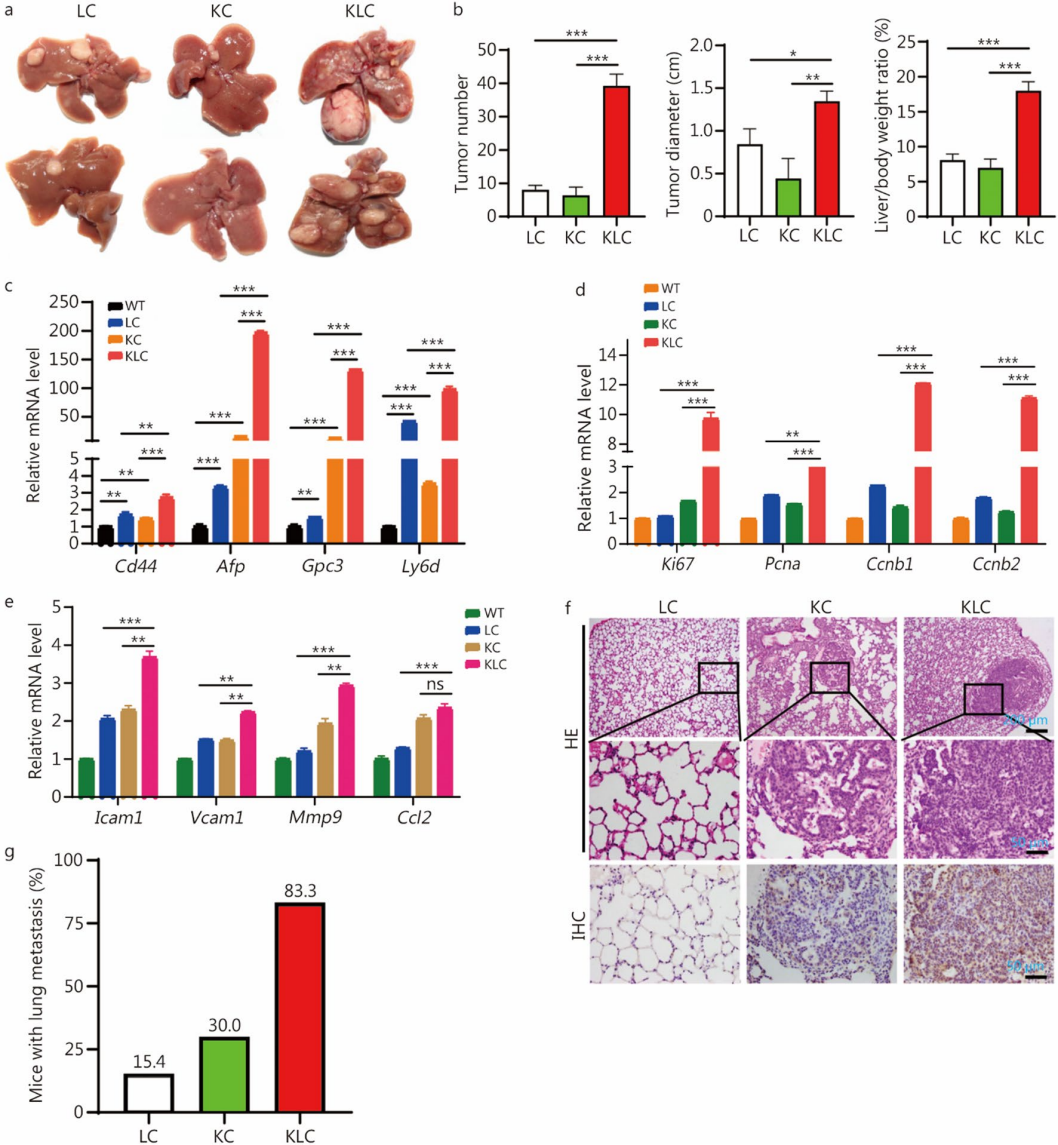
FBXL6 facilitates KRAS^G12D^-driven hepatocarcinogenesis and lung metastasis. *LSL-Fbxl6*^*KI/*+^*;Alb-Cre* (LC), *LSL-Kras*^*G12D/*+^*;Alb-Cre* (KC) and *LSL-Kras*^*G12D/*+^*;LSL-Fbxl6*^*KI/*+^*;Alb-Cre* (KLC) mice were monitored for 320 d and then sacrificed. **a** Representative images of tumorigenesis in LC, KC and KLC mice. **b** Quantification of the tumor number, tumor size, and liver/body weight ratio of LC (*n* = 13), KC (*n* = 10), and KLC mice (*n* = 12). qPCR was utilized to measure the expression of HCC markers (*Cd44*, *Afp*, *Gpc3* and *Ly6d*) (**c**), proliferation markers (*Ki67*, *Pcna*, *Ccnb1*, and *Ccnb2*) (**d**), and metastasis-related markers (*Icam1*, *Vcam1*, *Mmp9* and *Ccl2*) (**e**), in WT liver tissues, LC tumors, KC tumors and KLC tumors. Lung tissues were collected from LC, KC and KLC mice for HE and IHC staining. Representative images of HE and IHC staining for lipase C (LIPC) showing distinct lung metastatic foci expressing LIPC (**f**). Scale bars = 200 or 50 μm. The ratio of lung metastasis in each cohort was calculated (**g**). Data are represented as the mean ± SEM. One-way ANOVA was used in (**b**–**e**). ns non‑significant; ^*^P < 0.05; ^**^P < 0.01; ^***^P < 0.001. Fbxl6 F-box and leucine-rich repeat 6, Kras kirsten rat sarcoma, Afp alpha fetoprotein, Gpc3 glypican 3, Ly6d lymphocyte antigen 6 family member D, Ki67 marker of proliferation Ki-67, Pcna proliferating cell nuclear antigen, Ccnb1 cyclin B1, Ccnb2 cyclin B2, Icam1 intercellular adhesion molecule 1, Vcam1 vascular cell adhesion molecule 1, Mmp9 matrix metallopeptidase 9, Ccl2 C–C motif chemokine ligand 2, IHC immunohistochemistry, SEM standard error of the mean

Next, we wondered whether elevated FBXL6 in hepatocytes synergizes with KRAS mutation (KRAS^G12D^) to drive HCC. To this end, we constructed the following transgenic mouse strains: KC (*LSL-Kras*^*G12D/*+^*;Alb-Cre*) and KLC (*LSL-Kras*^*G12D/*+^*;LSL-Fbxl6*^*KI/*+^*;Alb-Cre*) mice (Additional file 1: Fig. S1a-d). *Alb-Cre* mice were used as the negative control (WT) unless otherwise stated. After 320 d of monitoring the incidence and development of HCC, we found that 100% of LC mice spontaneously developed HCC. Moreover, we observed that the KLC mouse subgroup developed many more, larger tumors and had a higher liver/body weight ratio than the KC and LC mouse groups (*P* < 0.05, Fig. 2a, b). Furthermore, compared with those of the LC or KC mouse group, the tumor tissues of the KLC mouse group exhibited elevated HCC markers (*Cd44, Afp, Gpc3 and Ly6d*) (*P* < 0.001 or *P* < 0.01, Fig. 2c). These results not only indicated that the KLC mice exhibited more severe liver damage than the LC and KC mice but also indicated the tumor-promoting role of Fbxl6. To further prove the oncogenic role of Fbxl6, we constructed *LSL-Kras*^*G12D/*+^*;CKO-Fbxl6*^*KO/*+^*;Alb-Cre* mice by crossing *LSL-Kras*^*G12D/*+^ mice with CKO-*Fbxl6*^*KO/*+^*;Alb-Cre* mice (Additional file 1: Fig. S3a-c). After 350 days of monitoring the development of HCC, we found that none of the *CKO-Fbxl6*^*KO/*+^*;Alb-Cre* mice spontaneously developed liver cancer (Additional file 1: Fig. S3d-e). Moreover, we observed that *LSL-Kras*^*G12D/*+^*;Alb-Cre* mice developed more severe hepatocarcinogenesis (tumor number, tumor size and the liver/body weight ratio) than *LSL-Kras*^*G12D/*+^*;CKO-Fbxl6*^*KO/*+^*;Alb-Cre* mice (*P* < 0.01, Additional file 1: Fig. S3d–e), suggesting that the knockout of *Fbxl6* attenuated Kras^G12D^-driven HCC tumorigenesis. Collectively, these results further strengthened the notion that Fbxl6 acts as an oncogene in HCC.

Previous studies demonstrated that the majority of HCCs (almost 90%) develop due to successive chronic liver inflammation, compensatory liver regeneration, and subsequent cirrhosis [25, 26]. Therefore, we detected the markers of proliferation and metastasis in WT, LC, KC and KLC mouse liver tissues using qPCR. The results showed that levels of the proliferation markers *Ki67, Pcna, Ccnb1 and Ccnb2* were remarkably elevated in KLC tumors compared with LC or KC tumors (*P* < 0.01 or *P* < 0.001, Fig. 2d). Moreover, the levels of metastasis markers (*Icam1, Vcam1, Mmp9 and Ccl2*) were also markedly increased in KLC tumors (*P* < 0.01 or *P* < 0.001, Fig. 2e). We further explored the lung metastasis of the above three cohorts of transgenic mice using HE and IHC staining. The data showed that KLC mice developed lung cancers that migrated from liver tumors, as indicated by high expression of the hepatocyte biomarker lipase C (LIPC) in lung tissues (Fig. 2f). The results showed that 15.4% (2/13) of the LC mice and 30.0% (3/10) of the KC mice developed lung metastasis. In contrast, 83.3% (10/12) of the KLC mice developed lung metastasis (Fig. 2g). Taken together, these results indicated that Fbxl6 facilitates Kras^G12D^-driven hepatocarcinogenesis and lung metastasis in vivo.

### FBXL6 activates the oncogenic KRAS/MEK/ERK axis and then promotes mTOR signaling activation

**Fig. 3:**
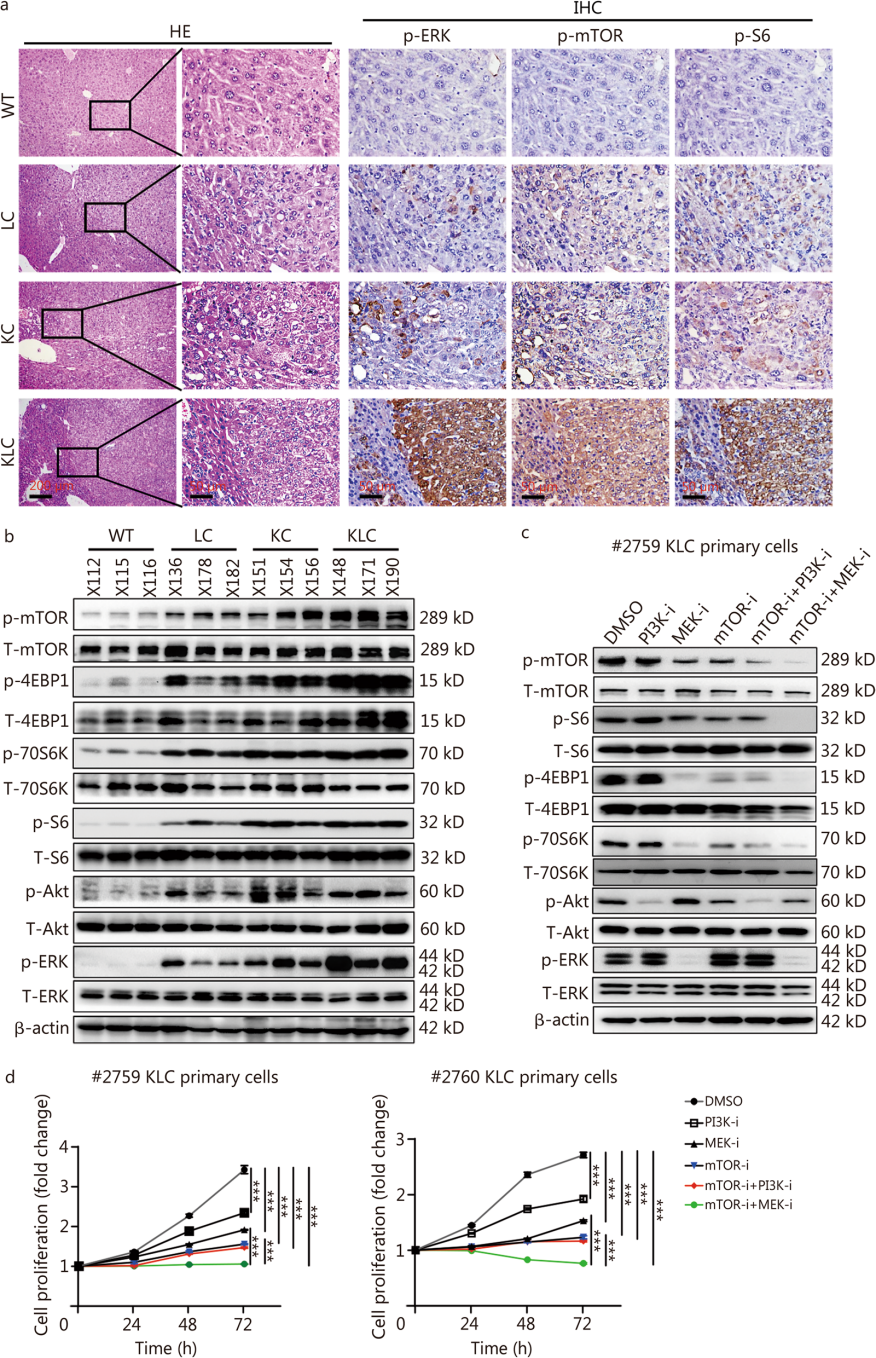
FBXL6 activates the oncogenic KRAS/MEK/ERK axis and promotes mTOR activation. **a** HE staining was utilized to determine the cancer tissue structure, and IHC was used to measure the p-ERK, p-mTOR, and p-S6 protein signals in WT, LC, KC and KLC mice. Representative consecutive IHC staining images are presented. Scale bars = 200 or 50 μm. **b** Western blotting was utilized to determine the protein levels of p-mTOR, p-4EBP1, p-70S6K, p-S6, p-Akt and p-ERK in WT (X112, 115 and 116), LC (X136, 178 and 182), KC (X151, 154 and 156) and KLC (X148, 171 and 190) mice. **c** After treatment with pharmacological inhibitors of PI3K (GDC-0326, 1 μmol/L), MEK (trametinib, 100 nmol/L), or mTOR (everolimus, 100 nmol/L); a PI3K inhibitor combined with an mTOR inhibitor; or an MEK inhibitor combined with an mTOR inhibitor for 48 h, KLC primary cells were lysed to extract total proteins. Thereafter, Western blotting was employed to measure the protein levels of p-mTOR, p-S6, p-4EBP1, p-70S6K, p-Akt and p-ERK. **d** After treatment with pharmacological inhibitors of PI3K (GDC-0326, 1 μmol/L), MEK (trametinib, 100 nmol/L), and mTOR (everolimus, 100 nmol/L) alone; a PI3K inhibitor combined with an mTOR inhibitor; or an MEK inhibitor combined with an mTOR inhibitor for 24, 48, and 72 h, the proliferation of KLC primary cells was analyzed by CCK-8 assay. Two-way ANOVA with the Bonferroni correction for multiple comparisons was used. ^***^P < 0.001. Fbxl6 F-box and leucine-rich repeat 6, Kras kirsten rat sarcoma, MEK mitogen-activated protein kinase kinase, ERK extracellular signal-regulated kinase, IHC immunohistochemistry, WT wild-type, LC *LSL-Fbxl6*^*KI/*+^*;Alb-Cre*, KC *LSL-Kras*^*G12D/*+^*;Alb-Cre*, KLC *LSL-Kras*^*G12D/*+^*;LSL-Fbxl6*^*KI/*+^*;Alb-Cre*, PI3K phosphatidylinositol 3‑kinase, mTOR mammalian target of rapamycin, S6 ribosomal protein S6, 70S6K ribosomal protein S6 kinase B1, 4EBP1 eukaryotic translation initiation factor 4E binding protein 1, PI3K-i inhibitor of PI3K, MEK-i inhibitor of MEK, mTOR-i inhibitor of mTOR

As the MAPK/ERK and PI3K/Akt signaling cascades are two main signaling pathways downstream of Kras, we explored which pathway is involved in Fbxl6- and Kras mutant-driven hepatocarcinogenesis by performing HE and IHC staining. The HE results verified the cancer tissue and normal tissue structure in mouse livers, and the IHC results showed that the phosphorylation of ERK, mTOR and S6 was markedly increased in KLC tumors compared with KC or LC tumors (Fig. 3a). We next analyzed the ERK and PI3K signaling pathways in liver tissues isolated from the WT (X112, X115 and X116), LC (X136, X178 and X182), KC (X151, X154 and X156) and KLC (X148, X171 and X190) mice using Western blotting. The results showed that p-mTOR, p-S6, p-4EBP1 and p-70S6K (the phosphorylation of Akt, ERK and mTOR signaling pathway-related proteins) were dramatically elevated in KLC tumors compared with either KC or LC tumors (Fig. 3b). Previous studies have demonstrated that the mTOR signaling pathway can be activated by both the MEK/ERK and PI3K/Akt cascades [27, 28]. Therefore, we further explored which pathway plays a key role in the activation of mTOR using KLC primary cells. As shown in Fig. 3c, inhibition of MEK, but not PI3K, significantly suppressed the activation of the mTOR cascade (p-mTOR, p-S6, p-4EBP1 and p-70S6K) in KLC primary cells. Furthermore, combinatorial inhibition of mTOR and MEK (but not mTOR and PI3K) almost completely inhibited the mTOR signaling pathway (p-mTOR, p-S6, p-4EBP1 and p-70S6K) in KLC primary cells (#2759). These results indicated that the MEK/ERK axis is the driving force behind FBXL6-mediated mTOR activation in HCC tumors with or without KRAS mutation. In addition, treatment with MEK-i (MEK inhibitor) and mTOR-i (mTOR inhibitor) dramatically suppressed the proliferation of KLC primary cells (#2759 and #2760) (*P* < 0.001, Fig. 3d). Taken together, these results indicate that FBXL6 activates the oncogenic KRAS/MEK/ERK axis, promoting mTOR signaling activation, which leads to the carcinogenesis and development of liver cancer. Moreover, dual inhibition of MEK and mTOR effectively suppressed the growth of this HCC cohort.

### Fbxl6 elevation synergizes with Kras^G12D^ to drive HCC by upregulating Prelid2

**Fig. 4:**
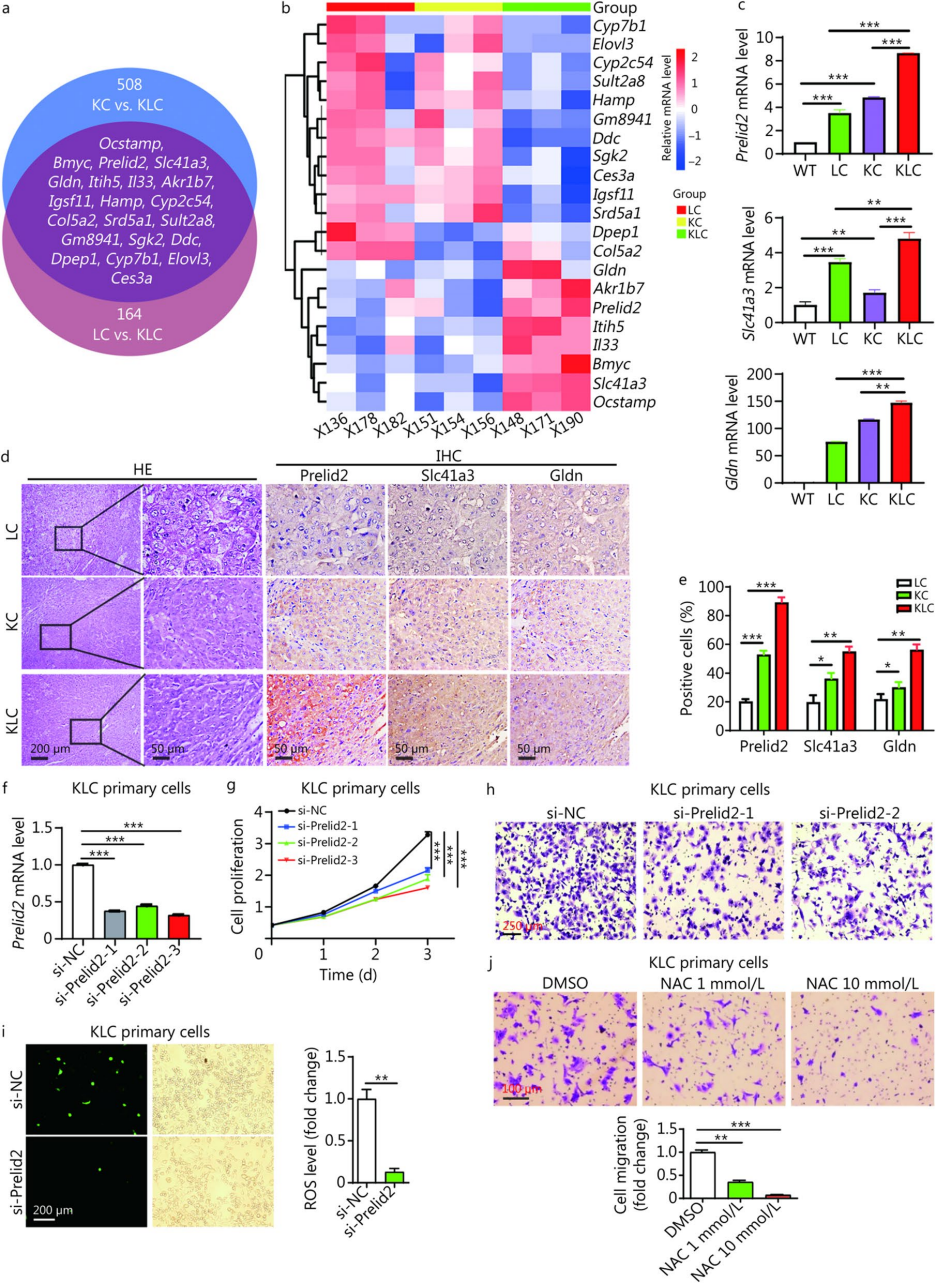
Fbxl6 elevation synergizes with Kras^G12D^ to drive HCC by upregulating Prelid2.** a** Venn diagram showing genes that were significantly differentially expressed at the mRNA level between liver tissues from KC and KLC mice and those from LC and KLC mice. **b** Heatmap showing the top 21 gene signatures representing different groups. **c** qPCR was used to detect the mRNA levels of *Prelid2*, *Slc41a3* and *Gldn* in WT, LC, KC and KLC mice. **d** HCC tissues were collected from LC, KC and KLC mice for HE and IHC staining. Representative consecutive IHC staining images for Prelid2, Slc41a3 and Gldn are presented. Scale bars = 200 or 50 μm. **e** Cells that were positive for Prelid2, Slc41a3, or Gldn were counted among a total of 500 cells on average from 3 independent tumors derived from 3 mice per group. **f** The *Prelid2* knockdown efficiency was determined by qPCR. Knockdown of *Prelid2* inhibited cell proliferation (**g**), migration (**h**), and ROS generation (**i**) in KLC primary cells. **j** Blockade of ROS with NAC (1 or 10 mmol/L) inhibited cell migration in KLC primary cells. Scale bars = 250 (**h**), 200 (**i**), or 100 (**j**) μm. Unpaired Student’s *t* test was used to analyze the data in (**i**). One-way ANOVA with Tukey’s multiple comparisons test was used to analyze the data in (**c**, **e**, **f**, **j**). ^*^P < 0.05; ^**^P < 0.01; ^***^P < 0.001. Fbxl6 F-box and leucine-rich repeat 6, Kras kirsten rat sarcoma, Prelid2 the proteins of relevant evolutionary and lymphoid interest (PRELI) domain 2, WT wild-type, LC *LSL-Fbxl6*^*KI/*+^;*Alb-Cre*, KC *LSL-Kras*^*G12D/*+^;*Alb-Cre*, KLC *LSL-Kras*^*G12D/*+^;*LSL-Fbxl6*^*KI/*+^;*Alb-Cre*, Slc41a3 solute carrier family 41 member 3, Gldn gliomedin, IHC immunohistochemistry, HCC hepatocellular carcinoma, ROS reactive oxygen species, NAC N-acetylcysteine

To identify the downstream effector in this subset of patients with FBXL6 elevation and KRAS activation, a panel of liver tumors from LC, KC and KLC mice were analyzed by transcriptome sequencing. The parameters for biomarker screening were set as follows: log_2_ (read count) > 1, log_2_ (fold change) > 2, and a statistical cut-off for the false discovery rate of < 0.05. A total of 508 genes were found to be differentially expressed between the KC and KLC cohorts, and 164 genes were differentially expressed between the LC and KLC cohorts, among which there were 21 overlapping genes with fragments per kilobase million (FPKM) values > 0.5 (Fig. 4a, b). To explore the functional relevance of these genes in HCC, we analyzed the overall survival (OS) of HCC patients using the TCGA database. Three genes (*PRELID2*, *SLC41A3* and *GLDN*) were significantly associated with the poor survival of HCC patients (*P* < 0.05). Moreover, qPCR was used to analyze the mRNA levels of these genes in the WT, LC, KC, and KLC mouse groups. The results showed that the expression of *Prelid2*, *Slc41a3* and *Gldn* was upregulated in KLC tumors compared with KC or LC tumors (*P* < 0.01, Fig. 4c). Additionally, the HE results verified the liver tissue structure, and IHC analysis revealed that the fold induction of the Prelid2 protein in KLC tumors was greater than that of Slc41a3 or Gldn (Fig. 4d, e). Interestingly, the amplitude of the Prelid2 protein level increased to a significantly greater extent than its mRNA level in KLC tumors compared with KC or LC tumors, suggesting that Prelid2 protein stability may be increased in KLC tumors (Fig. 4c, e). Previous studies have demonstrated that TRIAP1 is highly expressed in HCC and that the TRIAP1/PRELID1 complex is essential for sustaining PRELID1 protein stability and activity [29, 30]. Therefore, we hypothesized that TRIAP1 may interact with PRELID2 and then enhance PRELID2 protein stability. A CHX pulse-chase assay revealed that the knockdown of *Triap1* shortened the half-life of Prelid2 (Additional file 1: Fig. S4a). Moreover, a Co-IP assay indicated that Triap1 interacted with Prelid2 (*P* < 0.001, Additional file 1: Fig. S4b). Furthermore, we found that an mTOR inhibitor dramatically decreased the mRNA levels of *Triap1* and *Prelid2* (*P* < 0.001, Additional file 1: Fig. S4c). Collectively, these results indicated that Triap1 binds Prelid2 and then enhances Prelid2 protein stability, which may contribute to the high level of Prelid2 activity in HCC.

Next, we utilized the TCGA database to validate the poor prognosis of HCC patients with high expression of PRELID2. As shown in Additional file 1: Fig. S5a, the mRNA levels of *PRELID2* were dramatically upregulated in 371 primary tumors compared with 50 normal tissues (*P* < 0.001). This result was further validated by 50 paired HCC and adjacent normal tissues from the TCGA database (*P* < 0.001, Additional file 1: Fig. S5b). Furthermore, a high level of PRELID2 was closely associated with tumor grade (*P* < 0.01 or *P* < 0.001, Additional file 1: Fig. S5c). Interestingly, we found that HCC patients with *P53* mutations exhibited a higher level of PRELID2 than patients with *P53* non-mutant (*P* < 0.001, Additional file 1: Fig. S5d), suggesting a tumor-promoting role of PRELID2 in HCC. In addition, HCC patients with high expression of PRELID2 had a shorter lifespan than patients with low expression of PRELID2 (*P* < 0.001), in contrast, PRELID1 was not correlated with a poor prognosis in HCC patients (*P* = 0.082, Additional file 1: Fig. S5e). Moreover, knockdown of *Prelid2* markedly inhibited the growth and migration of KLC primary cells, as revealed by decreased cell proliferation and migrated cells (*P* < 0.001, Fig. 4f–h). Additionally, we determined the tumor-promoting role of Prelid2 in vivo by constructing KLC primary cells with stable *Prelid2* knockdown (LV-shPrelid2) (*P* < 0.001, Additional file 1: Fig. S6a). In line with the in vitro cell assays, we found *Prelid2* knockdown markedly decreased the tumor volume and weight of HCC xenograft (*P* < 0.01, Additional file 1: Fig. S6b-f), indicating that Fbxl6 elevation synergizes with Kras^G12D^ to drive HCC tumorigenesis via the upregulation of Prelid2. Thereafter, we explored the mechanism by which Prelid2 promotes HCC. Prelid2 belongs to the Preli-like family of proteins, which are mainly involved in mitochondrial lipid transport and mitochondrial ROS generation. Therefore, we determined whether Prelid2 influenced ROS production. The results showed that knockdown of *Prelid2* dramatically suppressed ROS generation in KLC primary cells (*P* < 0.01, Fig. 4i). Inhibition of ROS by the antioxidant N-acetylcysteine (NAC, 1 or 10 mmol/L) markedly repressed Fbxl6- and Kras mutant-mediated migration in KLC cells (*P* < 0.01 or *P* < 0.001, Fig. 4j). These results indicated that Prelid2 is a key downstream effector of HCC with high Fbxl6 expression and Kras activation and promotes the growth of cancer cells via ROS generation.

### mTOR and MEK inhibitors significantly blocked hepatocarcinogenesis and lung metastasis triggered by Fbxl6 elevation and *Kras* mutation

**Fig. 5:**
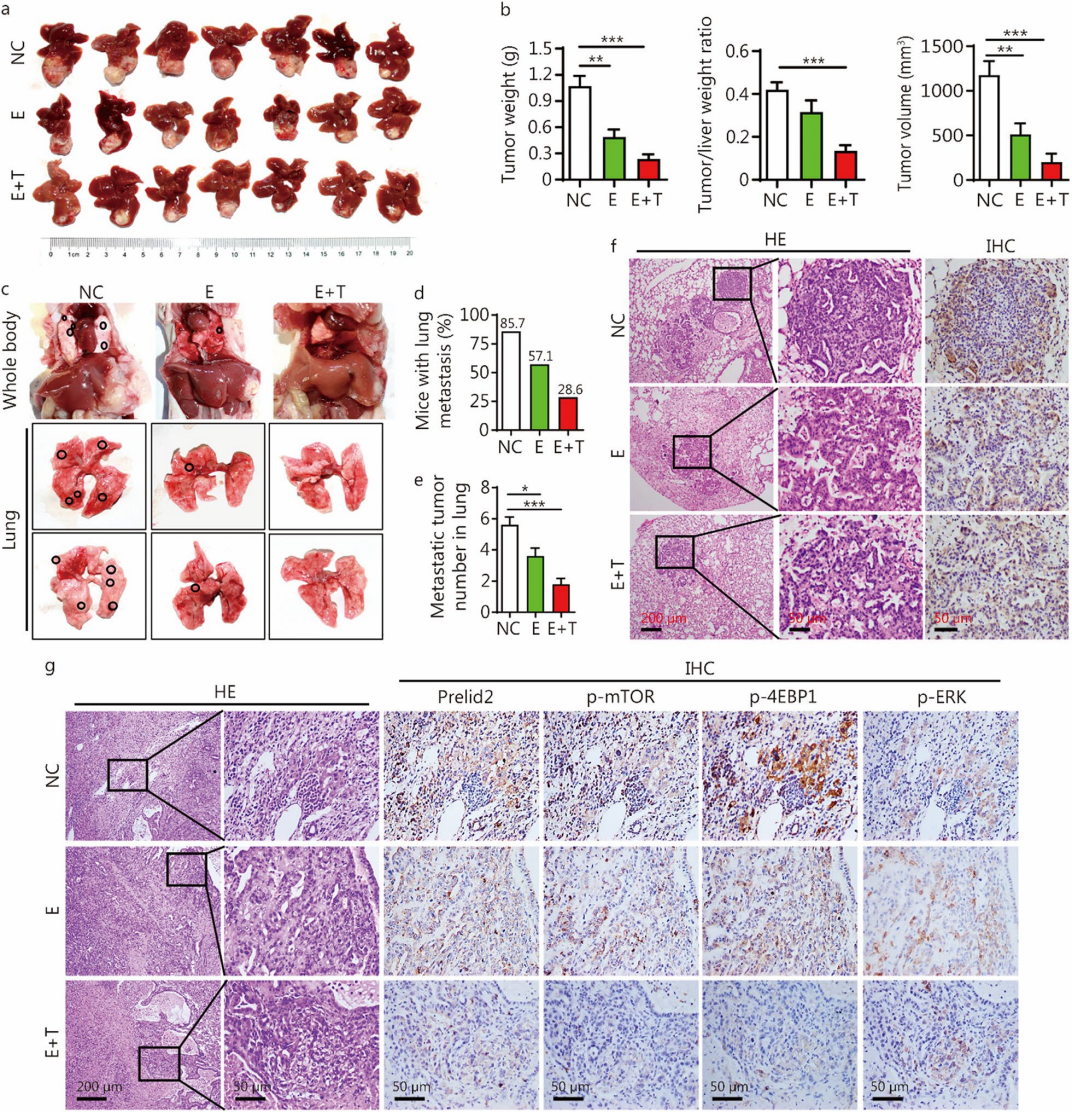
mTOR and MEK inhibition significantly blocks hepatocarcinogenesis and lung metastasis triggered by Fbxl6 elevation and Kras mutation. **a** Nude mice bearing KLC orthotopic HCC tumors were randomized into three groups: the negative control (NC) group, everolimus (E) group and everolimus combined with trametinib (E + T) group. The NC group was treated with vehicle, the E group was treated with everolimus (diluted in DMSO and coil oil, 4 mg/kg, intraperitoneal injection) twice per week for six rounds, and the E + T group was treated with both everolimus (the everolimus dose was as in the E group) and trametinib (diluted in DMSO and coil oil, 0.5 mg/kg, intraperitoneal injection) twice per week for six rounds. The mice were sacrificed after the last injection, and representative images showing tumorigenesis are presented. *n* = 7. **b** Tumor weight, the tumor/liver weight ratio, and tumor volume were analyzed (*n* = 7). **c** Representative images showing the effect of mTOR and MEK/ERK inhibitors on lung metastases. The lung metastasis rate (**d**) and number of lung distant lung metastatic foci (**e**) were quantified. **f** Representative HE and IHC staining images for lipase C (LIPC) showing distinct lung metastatic foci expressing LIPC. Scale bars = 200 or 50 μm. **g** Representative images of HE and IHC staining for Prelid2, p-mTOR, p-4EBP1, and p-ERK in the NC, E and E + T groups. Representative consecutive IHC staining images are presented. Scale bars = 200 or 50 μm. One-way ANOVA with Tukey’s multiple comparisons test was used in (**b**, **e**). ^*^P < 0.05; ^**^P < 0.01; ^***^P < 0.001. KLC *LSL-Kras*^*G12D/*+^;*LSL-Fbxl6*^*KI/*+^;A*lb-Cre*, mTOR mammalian target of rapamycin, MEK mitogen-activated protein kinase kinase, Fbxl6 F-box and leucine-rich repeat 6, Kras kirsten rat sarcoma, NC negative control, E everolimus, T trametinib, DMSO dimethyl sulfoxide, ERK extracellular signal-regulated kinase, IHC immunohistochemistry

Since HCC with FBXL6^high^/KRAS^G12D^ (KRAS hyperactivation) was defined as a malignant subtype of HCC, we further explored a potential therapeutic strategy for this subtype of patients. A mouse model of orthotopic tumor xenografts derived from KLC primary cells was established, and then the mice were treated with the FDA-approved mTOR inhibitor everolimus (E) alone or in combination with the MEK inhibitor trametinib (E + T). The results showed that everolimus significantly reduced the tumor weight and tumor volume compared with those in NC group (*P* < 0.01, Fig. 5a, b). This effect was further enhanced when everolimus was administered in combination with trametinib (Fig. 5a, b). Moreover, HE assay and IHC staining revealed that everolimus remarkably suppressed the lung metastasis of liver cancer (57.1%, 4/7) than vehicle (85.7%, 6/7), which was further improved by additional treatment with trametinib (28.6%, 2/7) (*P* < 0.05, Fig. 5c–e). In line with these results, LIPC protein level was found to be downregulated in the lung metastasis of liver cancer after treatment with everolimus, which was enhanced by trametinib supplement (Fig. 5f), indicating that mTOR and MEK inhibitors significantly repressed HCC lung metastasis. To confirm that whether the ERK/mTOR/Prelid2 pathway was inhibited in vivo after treatment with everolimus alone or in combination with trametinib, IHC and HE staining were performed. As shown in Fig. 5g, everolimus markedly reduced the protein levels of Prelid2, p-mTOR and p-4EBP1, which was further enhanced by combination with trametinib. Collectively, these results indicated that dual inhibition of MEK and mTOR may be an effective therapeutic strategy for HCC patients with high FBXL6 expression and KRAS activation.

### Elevated PRELID2 is positively correlated with the FBXL6/p-ERK/p-mTOR pathway and a poor prognosis in HCC

**Fig. 6:**
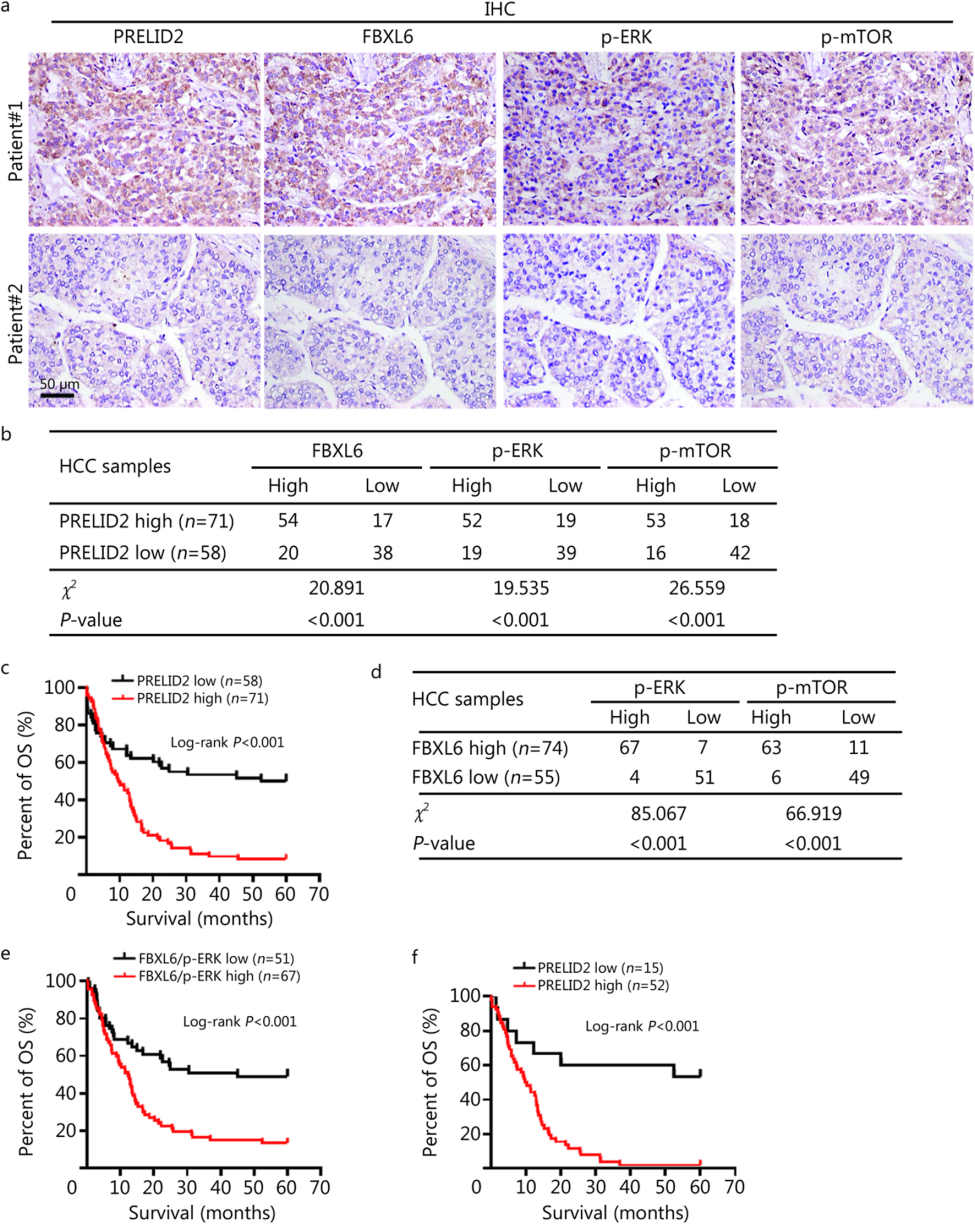
Elevated PRELID2 is positively correlated with the FBXL6/p-ERK/p-mTOR pathway and poor prognosis of HCC.** a** Representative images of IHC staining for FBXL6, PRELID2, p-ERK, and p-mTOR in human HCC tumors. Scale bar = 50 µm. **b** The association between PRELID2 and FBXL6, p-ERK, or p-mTOR in 129 paired HCC tumors and adjacent normal tissues was analyzed by *χ*^*2*^ test. **c** The prognostic significance of PRELID2 in HCC patients was evaluated by Kaplan–Meier analysis. High expression of PRELID2 predicted a shorter overall survival (OS) time. **d** The association between FBXL6 and p-ERK/p-mTOR protein levels in 129 HCC tissues was evaluated by the *χ*^*2*^ test. **e** The prognostic significance of the coexpression of FBXL6 and p-ERK in HCC patients was evaluated by Kaplan–Meier analysis. **f** Kaplan–Meier survival curves showing the overall survival of FBXL6^high^/p-ERK^high^ HCC patients with high or low PRELID2 expression. PRELID2 the proteins of relevant evolutionary and lymphoid interest (PRELI) domain 2, FBXL6 F-box and leucine-rich repeat 6, ERK extracellular signal-regulated kinase, mTOR mammalian target of rapamycin, IHC immunohistochemistry, HCC hepatocellular carcinoma

Since Prelid2 has been demonstrated to be a key downstream effector of MEK/ERK/mTOR-driven liver cancer in response to FBXL6 elevation and KRAS activation, we further evaluated the associations among PRELID2, FBXL6, p-ERK and p-mTOR in 129 paired human HCC and adjacent normal liver tissues from our department. IHC staining results indicated that high expression of PRELID2 was consistently correlated with elevated FBXL6 (*χ*^*2*^ = 20.891, *P* < 0.001), p-ERK (*χ*^2^ = 19.535,* P* < 0.001), and p-mTOR (*χ*^2^ = 26.559, *P* < 0.001) levels in HCC tumors (Fig. 6a, b). Furthermore, we found that PRELID2 protein levels were elevated in 55.0% (71/129) of the HCC samples compared with adjacent normal tissues (Fig. 6a, Additional file 1: Table S6). Moreover, PRELID2 was positively correlated with TNM stage (*P* < 0.001), tumor size (*P* = 0.013), tumor metastasis (*P* < 0.01), vascular thrombosis (*P* < 0.001) and recurrence (*P* < 0.001) (Additional file 1: Table S6). Consistent with these findings, PRELID2 was associated with UICC stage (*P* < 0.001), tumor stage (*P* < 0.001), tumor state (*P* = 0.019) and vascular invasion (*P* = 0.005) according to analysis of the TCGA dataset (Additional file 1: Table S7). Furthermore, a higher PRELID2 expression level was correlated with shorter OS in 129 HCC patients (log-rank *P* < 0.001, Fig. 6c). Both univariate and multivariate Cox survival analyses suggested that PRELID2 (*HR* = 0.361, 95%CI 0.230–0.568 *P* < 0.001; *HR* = 0.546, 95%CI 0.325–0.915, *P* = 0.022) and TNM stage (*HR* = 3.512, 95%CI 2.220–5.556, *P* < 0.001; *HR* = 2.268, 95%CI 1.230–4.181, *P* = 0.009, Additional file 1: Table S8) were independent risk factors for HCC.

We also found that a high level of FBXL6 was positively associated with the protein levels of p-ERK (*χ*^2^ = 85.067, *P* < 0.001) and p-mTOR (*χ*^2^ = 66.919, *P* < 0.001) in approximately 50% of HCC patients (Fig. 6a, d), which further suggested that the ERK/mTOR axis is a downstream target of FBXL6. Importantly, Kaplan–Meier analysis showed that patients with high FBXL6 and p-ERK coexpression in HCC tissues had a more severe prognosis than patients with low coexpression of these two proteins (log-rank *P* < 0.001, Fig. 6e). Consistently, high coexpression of FBXL6 and p-ERK was associated with a high TNM stage (*P* < 0.001), tumor size (*P* = 0.029), vascular thrombosis (*P* < 0.001), metastasis (*P* = 0.002) and outcome (*P* < 0.001, Additional file 1: Table S9). Importantly, univariate and multivariate Cox survival analyses suggested that high coexpression of both FBXL6 and p-ERK (*HR* = 0.409, 95%CI 0.255–0.653, *P* < 0.001; *HR* = 0.584, 95%CI 0.346–0.987, *P* = 0.044), TNM stage (*HR* = 3.506, 95%CI 2.161–5.688, *P* < 0.001; *HR* = 2.041, 95%CI 1.076–3.870, *P* = 0.029), and recurrence (*HR* = 4.431, 95%CI 2.262–8.682, *P* < 0.001; *HR* = 3.436, 95%CI 1.672–7.059, *P* < 0.001) were independent factors for OS in HCC patients (Additional file 1: Table S10). Kaplan–Meier analysis showed that patients with high PRELID2 expression had a poorer prognosis than patients with low PRELID2 expression (log-rank *P* < 0.001, Fig. 6f). Collectively, these results indicated that PRELID2 is positively associated with FBXL6 and p-ERK expression as well as poor prognosis in HCC patients.

## Discussion

In the current study, we revealed that elevated FBXL6 expression facilitates KRAS^G12D^-mediated induction of MEK/ERK/mTOR signaling via polyubiquitination-dependent activation of KRAS/KRAS^G12D^, leading to liver tumorigenesis and metastasis. The oncogenic activity of the MEK/ERK/mTOR axis relies on PRELID2. Dual inhibition of MEK and mTOR dramatically blocked FBXL6- and KRAS activation-driven HCC, and this information may provide a potential targeted approach for treating this cohort of HCC patients.

RAS proteins function as a molecular switch, receiving signals from numerous extracellular inputs and relaying them to an array of intracellular effectors. RAS is activated at the membrane downstream of a wide variety of cell-surface receptors, such as growth factor receptors [31], integrins [32], G-protein coupled receptors [33], and immune receptors [34]. Activated Ras transmits signals to its downstream pathways, including the RAF, PI3K-Akt-mTOR, Ral guanine dissociation stimulator (GDS), and phospholipase C (PLC) pathways, as well as many others [35, 36]. In this study, we found that KRAS^G12D^ preferentially activates mTOR via the MEK/ERK cascade in the context of high FBXL6 expression. Previous studies have revealed that FBXL6 is upregulated in many types of human cancers, such as CRC [19], renal cell carcinoma [37], and gastric cancer [38], and that FBXL6 upregulation contributes to tumorigenesis and cancer development. Consistent with these findings, we demonstrated that FBXL6 is dramatically elevated in HCC and is positively correlated with the poor prognosis of HCC patients. It is well known that HCC is a kind of cancer mainly caused by HBV or HCV infection, alcohol abuse, autoimmune hepatitis, diabetes mellitus, obesity, and several metabolic diseases [2, 3, 39, 40]. It would be very interesting to study which of these risk factors leads to the upregulation of FBXL6. Meanwhile, considering the high expression rate of FBXL6 in cancer, there is considerable interest in developing therapeutics that target cancers with high FBXL6 expression.

RAS is one of the most frequently mutated oncogenes in human cancers but is only rarely mutated in HCC [13, 28, 41, 42], suggesting that RAS mutation may not be the major factor contributing to RAS signaling activation. Recently, accumulating evidence has shown that the posttranslational regulation (especially ubiquitination) of RAS plays an important role in controlling its abundance and activity. Jeong et al. [17] reported that WDR76, a CUL4-DDB1 ubiquitin E3 ligase-interacting protein, degrades RAS via polyubiquitination-dependent proteasomal degradation, leading to suppression of the proliferation, transformation, motility, and invasive properties of HCC cells. Furthermore, the leucine zipper-like transcriptional regulator 1 (LZTR1) protein, an adaptor for the cullin3 (CUL3) ubiquitin ligase complex, was demonstrated to be an E3 linker protein and promote the ubiquitination of RAS at K170, inhibiting RAS signaling by attenuating its association with the membrane [22]. Interestingly, we observed that FBXL6 activated KRAS by enhancing its ubiquitination at K128. Although the ubiquitination of KRAS/NRAS at K128 and K147 was reported in a previous study [22], the corresponding E3 ligase has remained unclear. Our study is the first to observe that FBXL6 is the E3 ligase that mediates the ubiquitination of KRAS at the site K128. These data advance the current understanding of the activation of the RAS signaling pathway.

PRELID2 was demonstrated to be a downstream target of MEK/ERK/mTOR signaling and to play a key role in KRAS^G12D^ mutation- and FBXL6 elevation-driven HCC tumorigenesis. The proteins of relevant evolutionary and lymphoid interest (PRELI) domain containing family contains 6 proteins, namely PRELID1 (PRELI domain containing 1), PRELID2, PRELID3A, PRELID3B, PRELID4A and PRELID4B. Among the 6 PRELI domain family proteins, PRELID1 is the most extensively studied member and is highly expressed in fetal liver germinal centers in humans [43]. The PRELI-like family proteins, which are mainly involved in mitochondrial lipid transport and mitochondrial ROS generation [44], play important roles in embryonic development, lymphocyte differentiation, apoptosis, lipid metabolism and cancer [45]. However, the function of PRELID2 in cancer is still not entirely clear. Liu et al. [46] reported that PRELID2 is associated with poor survival in HNSCC patients and may be involved in radiotherapy resistance in nasopharyngeal carcinoma. Another study found that PRELID2 can be targeted and inhibited by miR-486-5p [47], which exerts a tumor-suppressive effect in several human cancers, including lung cancer [48], prostate cancer [49], and CRC [50]. In our study, we have revealed for the first time the tumor-promoting effect of PRELID2 in HCC. Elevated PRELID2 levels were positively correlated with the poor survival of HCC patients, which may support the development of PRELID2 as a target for HCC with elevated FBXL6 and activated KRAS. Although we found that the KRAS/MEK/mTOR signaling pathway regulates PRELID2 expression, the corresponding mechanism is still not known and needs to be further studied. Moreover, our results showed that PRELID2 promotes the proliferation and metastasis of liver cancer cells by enhancing ROS generation, which is similar to the function of PRELID1 in increasing ROS production [51].

**Fig. 7:**
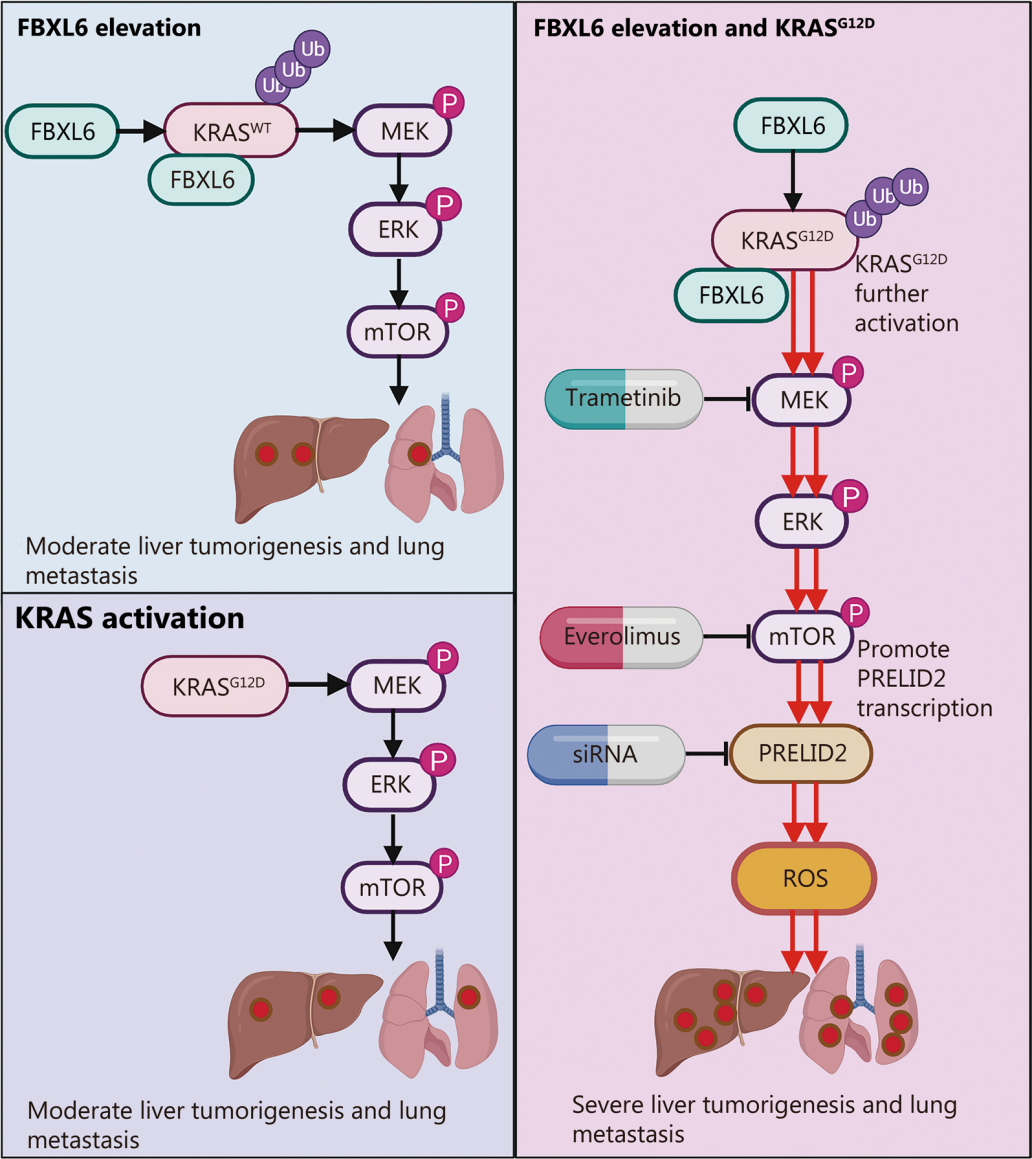
Working model of KRAS/KRAS^G12D^-triggered HCC in response to high FBXL6 expression. FBXL6 elevation facilitates the KRAS/KRAS^G12D^ mutation-mediated activation of MEK/ERK/mTOR signaling by promoting K63-linked KRAS/KRAS^G12D^ polyubiquitination at the site K128. Hyperactive mTOR increases the expression of PRELID2, leading to HCC tumorigenesis and metastasis in mice. This picture was created by BioRender. FBXL6 F-box and leucine-rich repeat 6, KRAS kirsten rat sarcoma, PRELID2 the proteins of relevant evolutionary and lymphoid interest (PRELI) domain 2, HCC hepatocellular carcinoma, MEK mitogen-activated protein kinase kinase, ERK extracellular signal-regulated kinase, mTOR mammalian target of rapamycin, WT wild-type, Ub ubiquitin, ROS reactive oxygen species

Collectively, our results demonstrate that FBXL6 elevation facilitates KRAS^G12D^-mediated activation of MEK/ERK/mTOR signaling by promoting the polyubiquitination of KRAS and KRAS^G12D^ at the site K128. Hyperactive mTOR increases the expression of PRELID2, leading to HCC tumorigenesis and metastasis in mice (Fig. 7). Interestingly, dual inhibition of MEK and mTOR dramatically represses hepatocarcinogenesis and lung metastasis and decreases the level of PRELID2, which may provide a potential strategy to treat this aggressive subtype of HCC with elevated FBXL6 and activated KRAS.

## Conclusions

In the current study, we reveal a previously unknown mechanism underlying KRAS activation induced by FBXL6-mediated ubiquitination. Specifically, FBXL6 promotes both WT KRAS and KRAS^G12D^ polyubiquitination at lysine 128, activating the MEK/ERK/mTOR/PRELID2/ROS signaling pathway, which drives tumorigenesis. Dual inhibition of MEK and mTOR effectively protects against FBXL6- and KRAS^G12D^-triggered hepatocarcinogenesis and lung metastasis, providing a potential strategy to treat this aggressive subtype of liver cancer.

### Supplementary Information

**Additional file 1**. **Fig. S1** Verification of mouse strains generation and PCR genotyping. **Fig. S2** FBXL6 enhances KRAS activity by K63-linked polyubiquitination. **Fig. S3** *Fbxl6* knockout counteract Kras^G12D^-driven hepatocarcinogenesis. **Fig. S4** Triap1 interacts with Prelid2 and enhances its protein stability. **Fig. S5** PRELID2 is a poor prognostic biomarker in HCC patients. **Fig. S6** Knockdown of *Prelid2* suppresses the growth of HCC xenograft tumors. **Table S1** PCR primers used for transgenic mice genotyping. **Table S2** PCR primers for site-directed mutagenesis. **Table S3** Sequences of siRNAs. **Table S4** Primers for qPCR. **Table S5** Sequences of shRNAs. **Table S6** Relationships between PRELID2 and clinicopathologic characteristics in 129 HCC patients of the IHC cohort [*n*(%)]. **Table S7** Relationships between PRELID2 and clinicopathologic characteristics of HCC patients in the 365 HCC patient of TCGA database [*n*(%)]. **Table S8** Univariate and multivariate analyses indicating associations between overall survival and various risk factors in the 129 HCC patients of IHC cohort. **Table S9** The relationship between co-expression of FBXL6/p-ERK and clinicopathological features in 118 HCC patients of IHC cohort [*n*(%)]. **Table S10** Univariate and multivariate analyses indicating associations between overall survival and various risk factors in the 118 HCC patients of IHC cohort.

## Data Availability

The data that support the findings of this study are available in the supplementary material of this article. The mass spectrometry proteomics data have been deposited to the ProteomeXchange Consortium (http://proteomecentral.proteomexchange.org) via the PRIDE partner repository with the dataset identifier PXD032357. The datasets and materials used or analyzed during the current study are available from the corresponding author on reasonable request.

## Abbreviations

Co-IP: Co-immunoprecipitation
CHX: Cycloheximide
CKO: Conditional knockout
DMSO: Dimethyl sulfoxide
ERK: Extracellular signal-regulated kinase
FBXL6: F-box and leucine-rich repeat 6
FBS: Fetal bovine serum
HCC: Hepatocellular carcinoma
IHC: Immunohistochemistry
KRAS: Kirsten rat sarcoma
LV: Lentivirus
mTOR: Mammalian target of rapamycin
NC: Negative control
PI3K: Phosphatidylinositol 3‑kinase
PRELID2: Proteins of relevant evolutionary and lymphoid interest (PRELI) domain 2
ROS: Reactive oxygen species
siRNA: Small interference RNA
BLCA: Bladder urothelial carcinoma
BRCA: Breast invasive carcinoma
CESC: Cervical squamous cell carcinoma and endocervical adenocarcinoma
CHOL: Cholangio carcinoma
COAD: Colon adenocarcinoma
ESCA: Esophageal carcinoma
GBM: Glioblastoma multiforme
HNSC: Head and neck squamous cell carcinoma
KICH: Kidney chromophobe
KIRC: Kidney renal clear cell carcinoma
KIRP: Kidney renal papillary cell carcinoma
LUAD: Lung adenocarcinoma
LUSC: Lung squamous cell carcinoma
PAAD: Pancreatic adenocarcinoma
PRAD: Prostate adenocarcinoma
PCPG: Pheochromocytoma and paraganglioma
READ: Rectum adenocarcinoma
SARC: Sarcoma
SKCM: Skin cutaneous melanoma
THCA: Thyroid carcinoma
THYM: Thymoma
STAD: Stomach adenocarcinoma
UCEC: Uterine corpus endometrial carcinoma
TCGA: The Cancer Genome Atlas
WCE: Whole cell extract
HA: Hemagglutinin
TP: Total protein
K128: Lysine 128
RBD: RAS-binding domain
AFP: Alpha fetoprotein
Gpc3: Glypican 3
Ly6d: Lymphocyte antigen 6 family member D
Ki67: Marker of proliferation Ki-67
Pcna: Proliferating cell nuclear antigen
Ccnb1: Cyclin B1
Ccnb2: Cyclin B2
Icam1: Intercellular adhesion molecule 1
Vcam1: Vascular cell adhesion molecule 1
Mmp9: Matrix metallopeptidase 9
Ccl2: C–C motif chemokine ligand 2
MEK: Mitogen-activated protein kinase kinase
S6: Ribosomal protein S6
70S6K: Ribosomal protein S6 kinase B1
4EBP1: Eukaryotic translation initiation factor 4E binding protein 1
PI3K-i: Inhibitor of PI3K
MEK-i: Inhibitor of MEK
mTOR-i: Inhibitor of mTOR
Slc41a3: Solute carrier family 41 member 3
Gldn: Gliomedin, HE staining hematoxylin–eosin staining
NAC: N-acetylcysteine

## References

[1] Villanueva A (2019). Hepatocellular carcinoma. N Engl J Med.

[2] Gu L, Zhu Y, Lin X, Lu B, Zhou X, Zhou F (2021). The IKKβ-USP30-ACLY axis controls lipogenesis and tumorigenesis. Hepatology.

[3] Zhu Y, Gu L, Lin X, Zhou X, Lu B, Liu C (2023). P53 deficiency affects cholesterol esterification to exacerbate hepatocarcinogenesis. Hepatology.

[4] Lu Y, Gao Y, Yang H, Hu Y, Li X (2022). Nanomedicine-boosting icaritin-based immunotherapy of advanced hepatocellular carcinoma. Mil Med Res.

[5] Craig AJ, von Felden J, Garcia-Lezana T, Sarcognato S, Villanueva A (2020). Tumour evolution in hepatocellular carcinoma. Nat Rev Gastroenterol Hepatol.

[6] Nault JC, Villanueva A (2015). Intratumor molecular and phenotypic diversity in hepatocellular carcinoma. Clin Cancer Res.

[7] Xu Y, Poggio M, Jin HY, Shi Z, Forester CM, Wang Y (2019). Translation control of the immune checkpoint in cancer and its therapeutic targeting. Nat Med.

[8] Cancer Genome Atlas Research Network (2017). Comprehensive and integrative genomic characterization of hepatocellular carcinoma. Cell.

[9] Misale S, Yaeger R, Hobor S, Scala E, Janakiraman M, Liska D (2012). Emergence of KRAS mutations and acquired resistance to anti-EGFR therapy in colorectal cancer. Nature.

[10] Jeong WJ, Yoon J, Park JC, Lee SH, Lee SH, Kaduwal S (2012). Ras stabilization through aberrant activation of Wnt/β-catenin signaling promotes intestinal tumorigenesis. Sci Signal..

[11] Zheng ZY, Tian L, Bu W, Fan C, Gao X, Wang H (2015). Wild-type N-Ras, overexpressed in basal-like breast cancer, promotes tumor formation by inducing IL-8 secretion via JAK2 activation. Cell Rep.

[12] Calaf GM, Abarca-Quinones J (2016). Ras protein expression as a marker for breast cancer. Oncol Lett.

[13] Calvisi DF, Ladu S, Gorden A, Farina M, Conner EA, Lee JS (2006). Ubiquitous activation of Ras and Jak/Stat pathways in human HCC. Gastroenterology.

[14] Chen L, Shi Y, Jiang CY, Wei LX, Wang YL, Dai GH (2011). Expression and prognostic role of pan-Ras, Raf-1, pMEK1 and pERK1/2 in patients with hepatocellular carcinoma. Eur J Surg Oncol.

[15] Yoshida T, Hisamoto T, Akiba J, Koga H, Nakamura K, Tokunaga Y (2006). Spreds, inhibitors of the Ras/ERK signal transduction, are dysregulated in human hepatocellular carcinoma and linked to the malignant phenotype of tumors. Oncogene.

[16] Dietrich P, Koch A, Fritz V, Hartmann A, Bosserhoff AK, Hellerbrand C (2018). Wild type kirsten rat sarcoma is a novel microRNA-622-regulated therapeutic target for hepatocellular carcinoma and contributes to sorafenib resistance. Gut.

[17] Jeong WJ, Park JC, Kim WS, Ro EJ, Jeon SH, Lee SK (2019). WDR76 is a RAS binding protein that functions as a tumor suppressor via RAS degradation. Nat Commun.

[18] Roukens MG, Alloul-Ramdhani M, Moghadasi S, Op Den Brouw M, Baker DA (2008). Downregulation of vertebrate Tel (ETV6) and Drosophila Yan is facilitated by an evolutionarily conserved mechanism of F-box-mediated ubiquitination. Mol Cell Biol.

[19] Li Y, Cui K, Zhang Q, Li X, Lin X, Tang Y (2021). FBXL6 degrades phosphorylated p53 to promote tumor growth. Cell Death Differ.

[20] Shi W, Feng L, Dong S, Ning Z, Hua Y, Liu L (2020). FBXL6 governs c-MYC to promote hepatocellular carcinoma through ubiquitination and stabilization of HSP90AA1. Cell Commun Signal.

[21] Sasaki AT, Carracedo A, Locasale JW, Anastasiou D, Takeuchi K, Kahoud ER (2011). Ubiquitination of K-Ras enhances activation and facilitates binding to select downstream effectors. Sci Signal.

[22] Steklov M, Pandolfi S, Baietti MF, Batiuk A, Carai P, Najm P (2018). Mutations in LZTR1 drive human disease by dysregulating RAS ubiquitination. Science.

[23] Baker R, Wilkerson EM, Sumita K, Isom DG, Sasaki AT, Dohlman HG (2013). Differences in the regulation of K-Ras and H-Ras isoforms by monoubiquitination. J Biol Chem.

[24] Komander D, Rape M (2012). The ubiquitin code. Annu Rev Biochem.

[25] Llovet JM, Zucman-Rossi J, Pikarsky E, Sangro B, Schwartz M, Sherman M (2016). Hepatocellular carcinoma. Nat Rev Dis Primers.

[26] Ringelhan M, Pfister D, O'Connor T, Pikarsky E, Heikenwalder M (2018). The immunology of hepatocellular carcinoma. Nat Immunol.

[27] Kong B, Wu W, Cheng T, Schlitter AM, Qian C, Bruns P (2016). A subset of metastatic pancreatic ductal adenocarcinomas depends quantitatively on oncogenic Kras/Mek/Erk-induced hyperactive mTOR signalling. Gut.

[28] Moore AR, Rosenberg SC, Mccormick F, Malek S (2020). RAS-targeted therapies: is the undruggable drugged?. Nat Rev Drug Discov.

[29] Ming M, Ying M, Ling M (2019). miRNA-125a-5p inhibits hepatocellular carcinoma cell proliferation and induces apoptosis by targeting TP53 regulated inhibitor of apoptosis 1 and Bcl-2-like-2 protein. Exp Ther Med.

[30] Potting C, Tatsuta T, König T, Haag M, Wai T, Aaltonen MJ (2013). TRIAP1/PRELI complexes prevent apoptosis by mediating intramitochondrial transport of phosphatidic acid. Cell Metab.

[31] Schlessinger J (2000). Cell signaling by receptor tyrosine kinases. Cell.

[32] Giancotti FG, Ruoslahti E (1999). Integrin signaling. Science.

[33] Dorsam RT, Gutkind JS (2007). G-protein-coupled receptors and cancer. Nat Rev Cancer.

[34] Huse M (2009). The T-cell-receptor signaling network. J Cell Sci.

[35] Molina-Arcas M, Hancock DC, Sheridan C, Kumar MS, Downward J (2013). Coordinate direct input of both KRAS and IGF1 receptor to activation of PI3 kinase in KRAS-mutant lung cancer. Cancer Discov.

[36] Simanshu DK, Nissley DV, McCormick F (2017). RAS proteins and their regulators in human disease. Cell.

[37] Yu Y, Yao W, Wang T, Xue W, Meng Y, Cai L (2022). FBXL6 depletion restrains clear cell renal cell carcinoma progression. Transl Oncol.

[38] Meng L, Hu YT, Xu AM (2023). F-box and leucine-rich repeat 6 promotes gastric cancer progression via the promotion of epithelial-mesenchymal transition. World J Gastrointest Oncol.

[39] Yang JD, Roberts LR (2010). Hepatocellular carcinoma: a global view. Nat Rev Gastroenterol Hepatol.

[40] Deng GH, Wu CF, Li YJ, Shi H, Zhong WC, Hong MK (2023). Caveolin-1 is critical for hepatic iron storage capacity in the development of nonalcoholic fatty liver disease. Mil Med Res.

[41] Turhal NS, Savaş B, Çoşkun Ö, Bas E, Karabulut B, Nart D (2015). Prevalence of K-Ras mutations in hepatocellular carcinoma: a Turkish Oncology Group pilot study. Mol Clin Oncol.

[42] Delire B, Stärkel P (2015). The Ras/MAPK pathway and hepatocarcinoma: pathogenesis and therapeutic implications. Eur J Clin Invest.

[43] Guzman-Rojas L, Sims JC, Rangel R, Guret C, Sun Y, Alcocer JM (2000). PRELI, the human homologue of the avian px19, is expressed by germinal center B lymphocytes. Int Immunol.

[44] Miliara X, Tatsuta T, Berry JL, Rouse SL, Solak K, Chorev DS (2019). Structural determinants of lipid specificity within Ups/PRELI lipid transfer proteins. Nat Commun.

[45] Zhu Y, Zou R, Sha H, Lu Y, Zhang Y, Wu J (2020). Lipid metabolism-related proteins of relevant evolutionary and lymphoid interest (PRELI) domain containing family proteins in cancer. Am J Transl Res.

[46] Liu G, Zeng X, Wu B, Zhao J, Pan Y (2020). RNA-Seq analysis of peripheral blood mononuclear cells reveals unique transcriptional signatures associated with radiotherapy response of nasopharyngeal carcinoma and prognosis of head and neck cancer. Cancer Biol Ther.

[47] Wang W, Ji J, Li J, Ren Q, Gu J, Zhao Y (2020). Several critical genes and microRNAs associated with the development of polycystic ovary syndrome. Ann Endocrinol.

[48] Wang J, Tian X, Han R, Zhang X, Wang X, Shen H (2014). Downregulation of miR-486-5p contributes to tumor progression and metastasis by targeting protumorigenic ARHGAP5 in lung cancer. Oncogene.

[49] Zhang X, Zhang T, Yang K, Zhang M, Wang K (2016). miR-486-5p suppresses prostate cancer metastasis by targeting Snail and regulating epithelial-mesenchymal transition. Onco Targets Ther.

[50] Liu C, Li M, Hu Y, Shi N, Yu H, Liu H (2016). miR-486-5p attenuates tumor growth and lymphangiogenesis by targeting neuropilin-2 in colorectal carcinoma. Onco Targets Ther.

[51] Tahvanainen J, Kallonen T, Lähteenmäki H, Heiskanen KM, Westermarck J, Rao KV (2009). PRELI is a mitochondrial regulator of human primary T-helper cell apoptosis, STAT6, and Th2-cell differentiation. Blood.

