# Supplementary material for: Elevated FBXL6 activates both wild-type KRAS and mutant KRASG12D and drives HCC tumorigenesis via the ERK/mTOR/PRELID2/ROS axis in mice
Source: Mil Med Res. 2023 Dec 20;10:68. doi: 10.1186/s40779-023-00501-8 (PMC10731709; doi:10.1186/s40779-023-00501-8)
Supplement: Supplementary file 1 — Additional file 1. Fig. S1 Verification of mouse strains generation and PCR genotyping. Fig. S2 FBXL6 enhances KRAS activity by K63-linked polyubiquitination. Fig. S3 Fbxl6 knockout counteract KrasG12D-driven hepatocarcinogenesis. Fig. S4 Triap1 interacts with Prelid2 and enhances its protein stability. Fig. S5 PRELID2 is a poor prognostic biomarker in HCC patients. Fig. S6 Knockdown of Prelid2 suppresses the growth of HCC xenograft tumors. Table S1 PCR primers used for transgenic mice genotyping. Table S2 PCR primers for site-directed mutagenesis. Table S3 Sequences of siRNAs. Table S4 Primers for qPCR. Table S5 Sequences of shRNAs. Table S6 Relationships between PRELID2 and clinicopathologic characteristics in 129 HCC patients of the IHC cohort [n(%)]. Table S7 Relationships between PRELID2 and clinicopathologic characteristics of HCC patients in the 365 HCC patient of TCGA database [n(%)]. Table S8 Univariate and multivariate analyses indicating associations between overall survival and various risk factors in the 129 HCC patients of IHC cohort. Table S9 The relationship between co-expression of FBXL6/p-ERK and clinicopathological features in 118 HCC patients of IHC cohort [n(%)]. Table S10 Univariate and multivariate analyses indicating associations between overall survival and various risk factors in the 118 HCC patients of IHC cohort. [file 40779_2023_501_MOESM1_ESM.pdf]

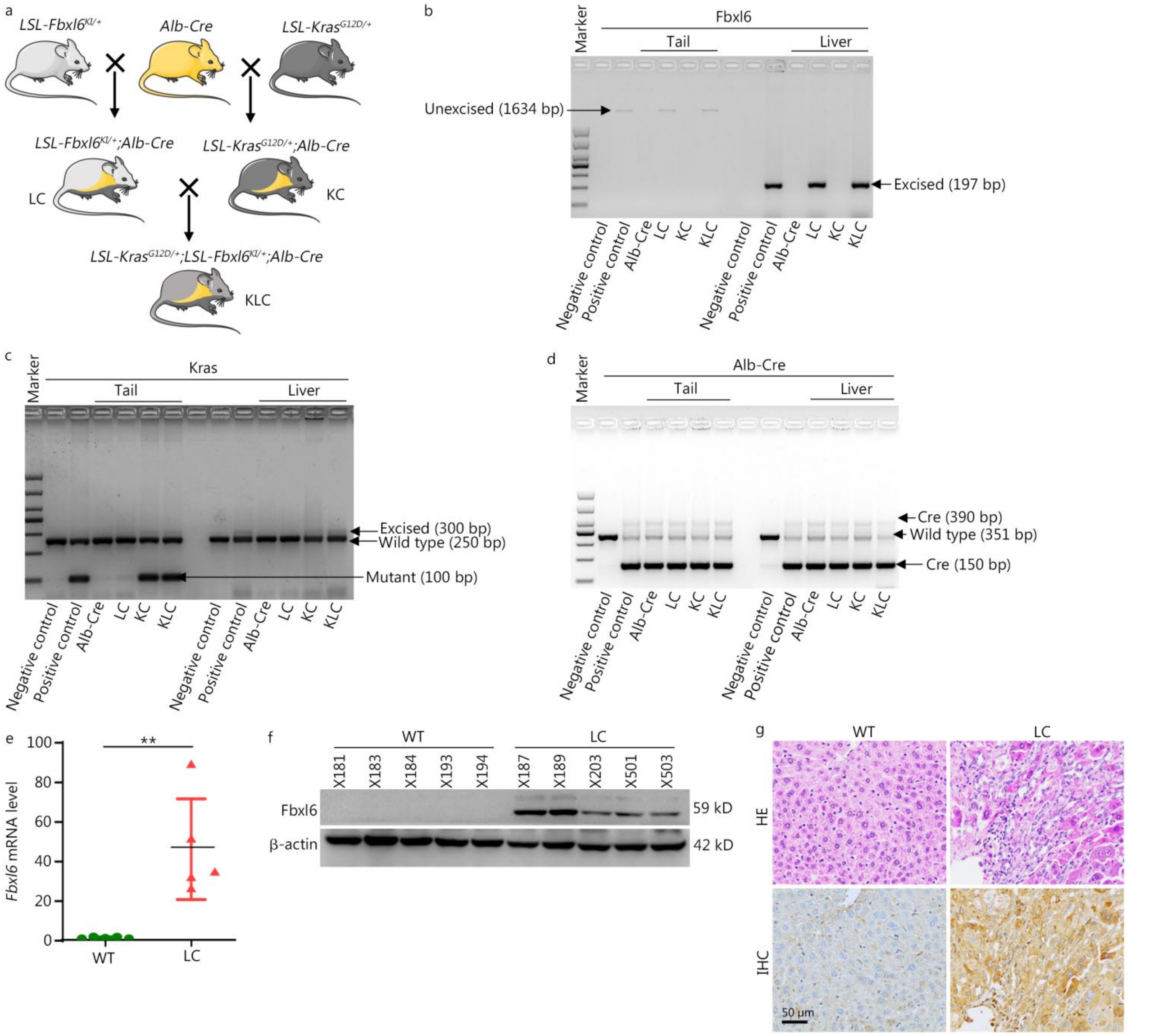

Fig. S1 Verification of mouse strains generation and PCR genotyping. **a** Mouse strains generation. *LSL-Kras*$^{G12D/+}$ mice were crossed with *Alb-Cre* mice to obtain *LSL-Kras*$^{G12D/+}$;*Alb-Cre* (KC) mice. *LSL-Fbxl6*$^{KI/+}$ mice were interbred with *Alb-Cre* mice to obtain *LSL-Fbxl6*$^{KI/+}$;*Alb-Cre* (LC) mice. KC mice were crossed with LC mice to generate *LSL-Kras*$^{G12D/+}$;*LSL-Fbxl6*$^{KI/+}$;*Alb-Cre* (KLC) mice. **b-d** PCR genotyping of mouse strains. DNA was extracted from the tail and liver tissues of *Alb-Cre* mice, LC, KC and KLC mice, followed with PCR genotyping. qPCR (**e**) and Western blotting (**f**) analysis of Fbxl6 expression at mRNA and protein levels in the liver of LC (X187, X189, X203, X501, X503 represent different mouse of same genotype, *n* = 5) and WT (X181, X183, X184, X193, X194 represent different mouse of same genotype, *n* = 5) mice. Unpaired *t*-test was used in **(e)**. **g** HE and IHC staining of Fbxl6 in the livers of LC (*n* = 5) and WT (*n* = 5) mice. Representative images of HE and IHC staining for Fbxl6 were shown. Scale bar = 50 μm. $^{**}P$ <0.01. PCR polymerase chain reaction, WT wild type, Fbxl6 F-box and leucine-rich repeat 6, Kras kirsten rat sarcoma, qPCR quantitative PCR

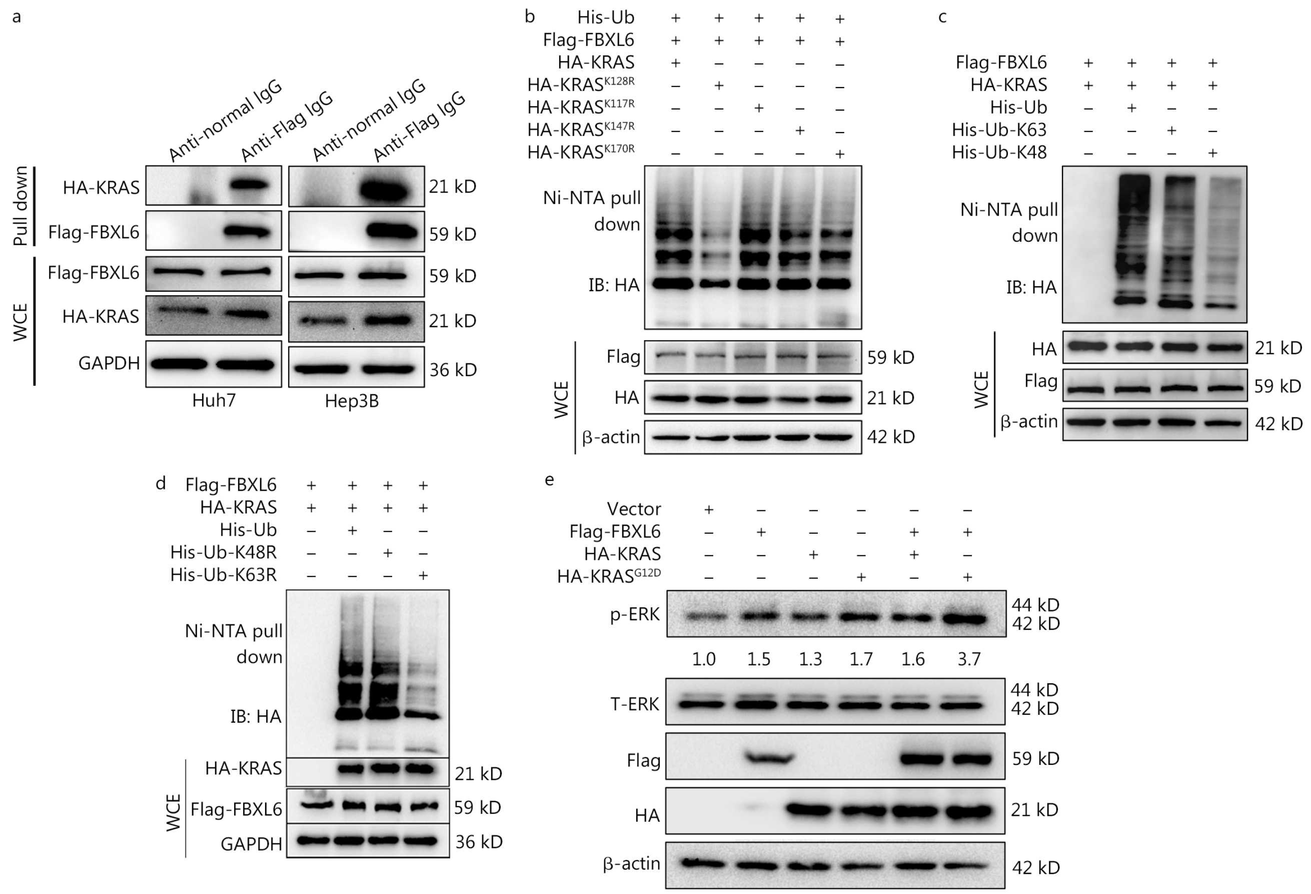

Fig. S2 FBXL6 enhances KRAS activity by K63-linked polyubiquitination. **a** Huh7 and Hep3B cells were transfected with indicated plasmids and lysed for immunoprecipitation with anti-Flag antibody or anti-mouse IgG antibody. **b** HEK293T cells were transfected with the indicated plasmids for 72 h, and then lysed with 6 mol/L guanidine solution, followed by pull-down using Ni-NTA beads or direct Western blotting analysis with the indicated antibodies. **c, d** Ubiquitin or its mutants (K63, K48, K63R, and K48R) plasmids were transfected into HEK293T cells along with FBXL6 and KRAS eukaryotic expression plasmids for 72 h, and then pulled down with Ni-beads, followed by Western blotting analysis with the indicated antibodies. **e** Huh7 cells were transfected with the indicated plasmids for 48 h, followed by Western blotting with the indicated antibodies. FBXL6 F-box and leucine-rich repeat 6, KRAS kirsten rat sarcoma, WCE whole-cell extract, IB immune blot, Ub ubiquitin, WT wild type, ERK extracellular signal-regulated kinase, GAPDH glyceraldehyde-3-phosphate dehydrogenase, HA hemagglutinin

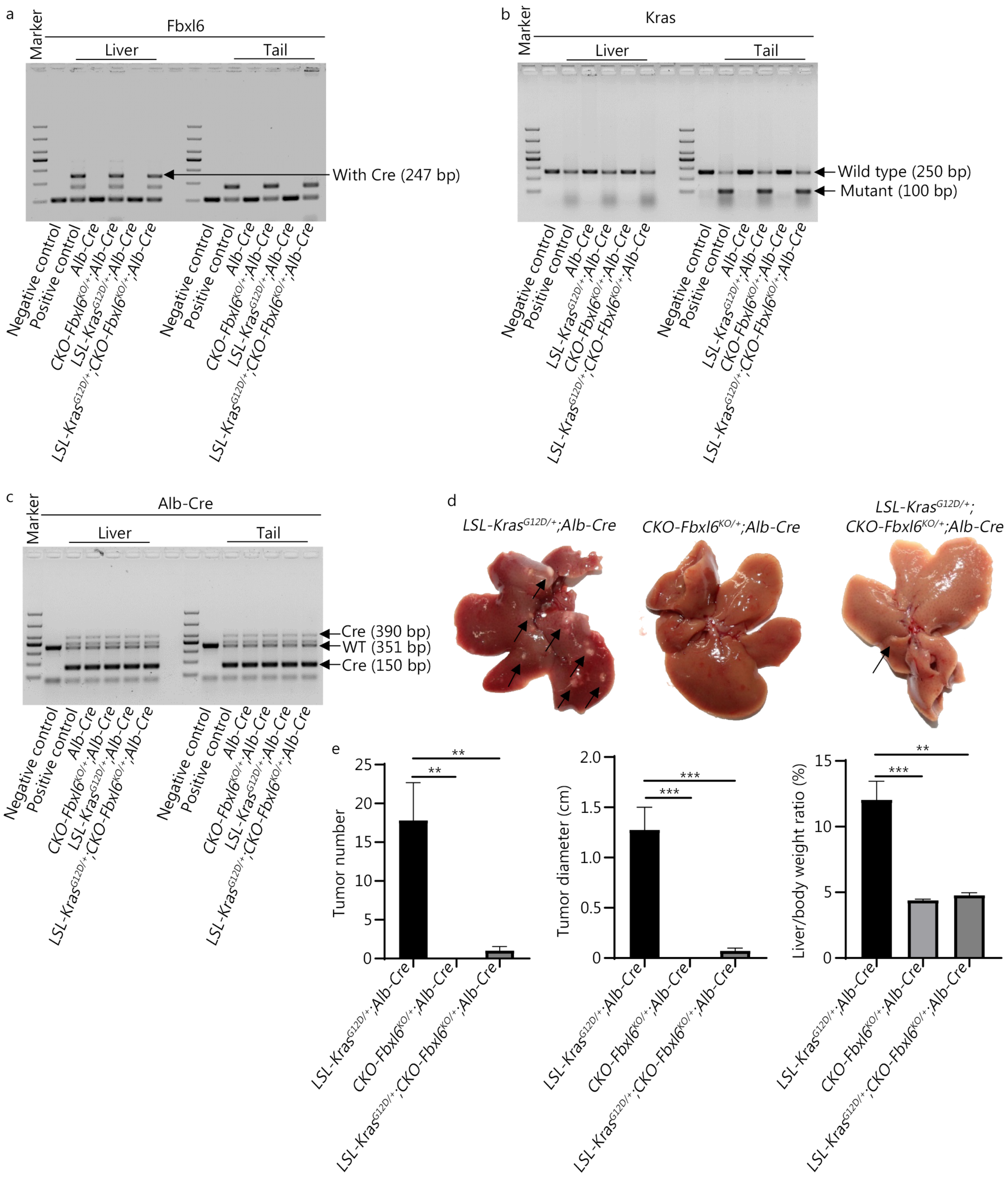

Fig. S3 *Fbxl6* knockout counteract $Kras^{G12D}$-driven hepatocarcinogenesis. **a-c** PCR genotyping of mouse strains. DNA was extracted from the tail and liver tissues of *CKO-Fbxl6*$^{KO/+}$*;Alb-Cre, LSL-Kras*$^{G12D/+}$*;Alb-Cre*, and *LSL-Kras*$^{G12D/+}$*;CKO-Fbxl6*$^{KO/+}$*;Alb-Cre* mice, followed with PCR genotyping. **d-e** *CKO-Fbxl6*$^{KO/+}$*;Alb-Cre, LSL-Kras*$^{G12D/+}$*;Alb-Cre*, and *LSL-Kras*$^{G12D/+}$*;CKO-Fbxl6*$^{KO/+}$*;Alb-Cre* mice were monitored more than 350 d, and then sacrificed. Representative tumorigenesis images were shown **(d)**. Quantification of the tumor number, largest tumor

size, and liver/body weight ratio in *LSL-Kras*$^{G12D/+}$*;Alb-Cre* (*n* = 5), *CKO-Fbxl6*$^{KO/+}$*;Alb-Cre* (*n* = 6), and *LSL-Kras*$^{G12D/+}$*;CKO-Fbxl6*$^{KO/+}$*;Alb-Cre* mice (*n* = 5) (**e**). One-way ANOVA was used in **(e)**. $^{**}P$ <0.01; $^{***}P$ <0.001. PCR polymerase chain reaction, WT wild type, Fbxl6 F-box and leucine-rich repeat 6, Kras kirsten rat sarcoma

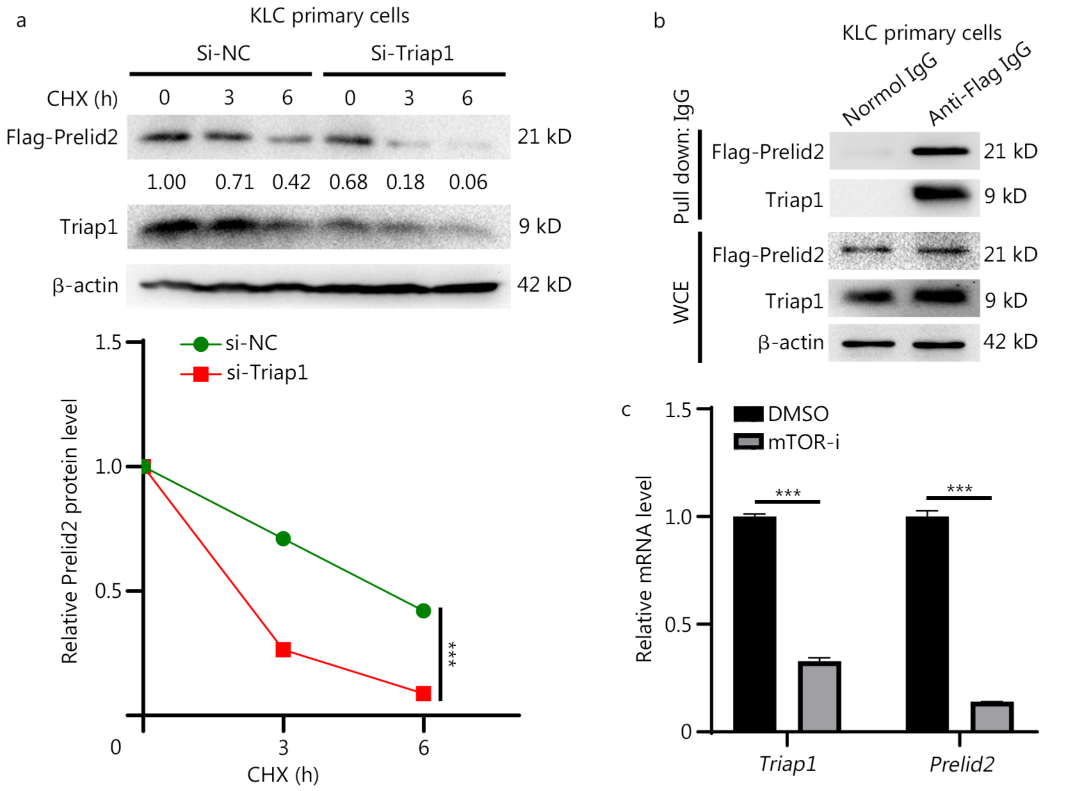

Fig. S4 Triap1 interacts with Prelid2 and enhances its protein stability. **a** KLC primary cells were transfected with Flag-Prelid2 expression plasmids for 48 h, followed by treatment with 100 μg/ml cycloheximide (CHX) for the indicated times, then lysed for Western blotting analysis. Band intensity was quantified by software ImageJ. β-actin was utilized as the internal control. **b** KLC primary cells were seeded in 10 cm dishes and transfected with Flag-Prelid2 plasmids for 48 h, followed by co-immunoprecipitated (Co-IP) assay using anti-Flag antibody or anti-mouse lgG (negative control) antibody. Western blotting was utilized to detect the indicated proteins. β-actin was used as the loading control. **c** After treatment with inhibitor of mTOR (everolimus, 100 nmol/L) for 48 h, KLC primary cells were collected for detecting *Triap1* and *Prelid2* mRNA levels by qPCR. β-actin was utilized as the internal control. $^{***}P < 0.001$. Triap1 TP53 regulated inhibitor of apoptosis 1, KLC *LSL-Kras*$^{G12D/+}$*;LSL-Fbxl6*$^{KI/+}$*;Alb-Cre*, NC negative control, si-Triap1 small interference RNA for Triap1, WCE whole-cell extract, mTOR-i inhibitor of mTOR

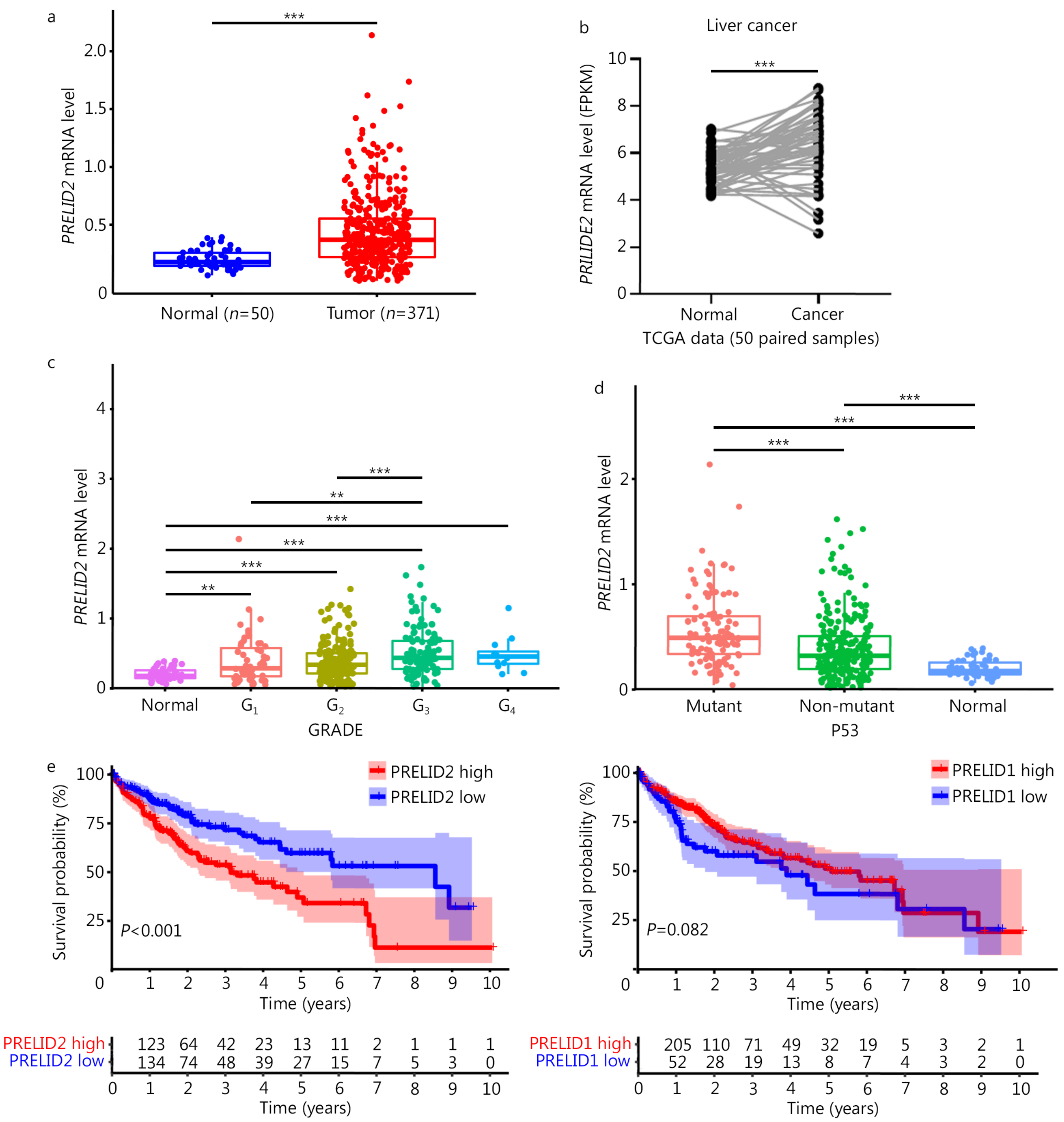

Fig. S5 PRELID2 is a poor prognostic biomarker in HCC patients. **a** The *PRELID2* mRNA expression of normal (n = 50) and HCC (n = 371) liver tissues in TCGA database. **b** The mRNA expression of *PRELID2* was analyzed in 50-paired HCC tumors and normal tissues in TCGA database. **c** The mRNA expression of *PRELID2* in HCC patients with different tumor grade. Normal (n = 50), G_1 (n = 54), G_2 (n = 173), G_3 (n = 118) and G_4 (n = 12). **d** The mRNA level of *PRELID2* in normal liver tissues and HCC tissues with *P53* mutation or not. Normal (n = 50), *P53* mutant (n = 105) and *P53* non-mutant (n = 255). **e** Kaplan-Meier analysis of the overall survival (OS) of HCC patients with high or low expression of PRELID2 or PRELID1. Unpaired t-test was used. $^{**}P < 0.01$; $^{***}P < 0.001$. PRELID2 the

proteins of relevant evolutionary and lymphoid interest (PRELI) domain 2, PRELID1 the proteins of relevant evolutionary and lymphoid interest (PRELI) domain 1, HCC hepatocellular carcinoma, TCGA The Cancer Genome Atlas

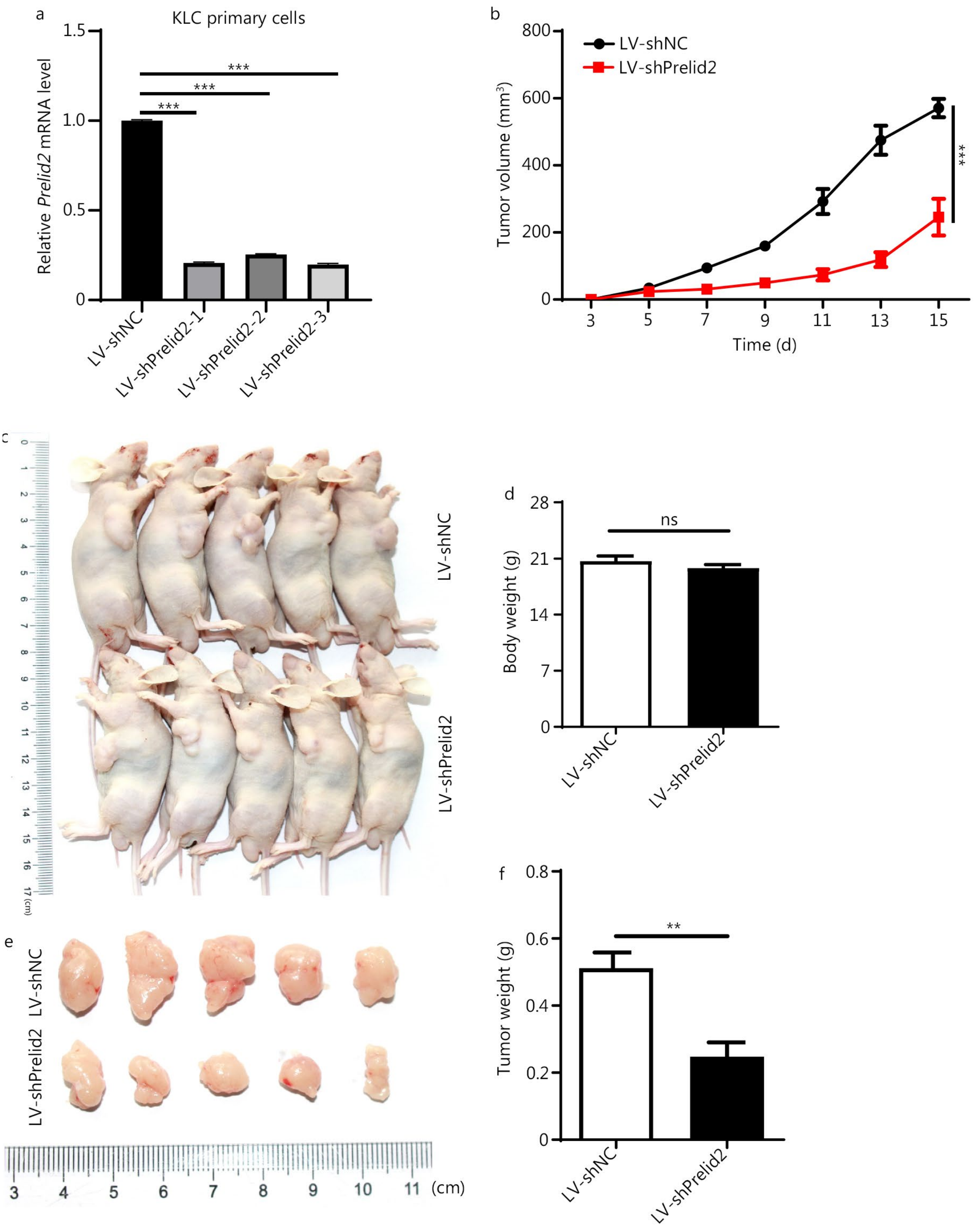

Fig. S6 Knockdown of *Prelid2* suppresses the growth of HCC xenograft tumors. **a** qPCR analysis showed the selected clones (*LSL-Kras*$^{G12D/+}$*;LSL-Fbxl6*$^{KI/+}$*;Alb-Cre*) stably expressing Prelid2-specific shRNA (LV-shPrelid2) or negative control shRNA (LV-shNC). **b-f** KLC cells (5×10^6) with or without *Prelid2* stable knockdown were separately injected into the right or left axilla of BALB/c nude mice of each group (n = 5 per group). Subsequently, the xenograft

tumor size was monitored every other day (volume = width^2 × length × 1/2) for 15 d **(b)**, then the mice were euthanatized and photographed **(c)**. Thereafter, the body weight of these mice was recorded **(d)**. After that, xenograft tumors were excised from the nude mice **(e)** and then weighted **(f)**. One-way ANOVA with Tukey's multiple comparisons test was used in **(b)**. Unpaired *t*-test was used in **(d, f)**. ns non-significant; $^{**}P < 0.01$; $^{***}P < 0.001$. Prelid2 the proteins of relevant evolutionary and lymphoid interest (PRELI) domain 2, KLC *LSL-Kras*$^{G12D/+}$*;LSL-Fbxl6*$^{KI/+}$*;Alb-Cre*, HCC hepatocellular carcinoma, qPCR quantitative PCR, LV lentivirus, shPrelid2 shRNA for Prelid2, shNC shRNA for negative control

Table S1 PCR primers used for transgenic mice genotyping

Gene	Primers (5' – 3')
LSL-Fbxl6$^{KI/+}$	Forward: CTGGGCAACGTGCTGGTTAT
	Reverse: CGACGTCTAACTCGCCTAGA
LSL-Kras^{G12D}	Wild type forward: TGTCTTTCCCCAGCACAGT
	Common: CTGCATAGTACGCTATACCCTGT
	Mutant forward: GCAGGTCGAGGGACCTAATA
Alb-Cre	Wild type forward: TGCAAACATCACATGCACAC
	Common: TTGGCCCCTTACCATAACTG
	Mutant forward: GAAGCAGAAGCTTAGGAAGATGG
CKO-Fbxl6$^{KO/+}$	Forward: GAGGAAGTCCAGTGGTGTCTAG
	Common: AAGTGAAAATGAGCAGGGTAAACC
	Reverse: GGTTGTAGTAAGACGCCGGCTAA

PCR polymerase chain reaction, *Fbxl6* F-box and leucine-rich repeat 6, *Kras* kirsten rat sarcoma

Table S2 PCR primers for site-directed mutagenesis

Gene	Forward (5' – 3')	Reverse (5' – 3')
$KRAS^{G12D}$	CTCTTGCCTACGCCATCAGCTCCAACTACCA	TGGTAGTTGGAGCTGATGGCGTAGGCAAGAG
$KRAS^{K128R}$	CTAAGTCCTGAGCCTGTCTTGTGTCTACTGTTCTAGA	TCTAGAACAGTAGACACAAGACAGGCTCAGGACTTAG
$KRAS^{K117R}$	CTAGAAGGCAAATCACATCTATTTCCTACTAGGACCATAGGTAC	GTACCTATGGTCCTAGTAGGAAATAGATGTGATTTGCCTTCTAG
$KRAS^{K147R}$	CCTCCACTCTCTGTCTTGTCCTTGCTGATGTTTCAATAAAAG	CTTTTATTGAAACATCAGCAAGGACAAGACAGAGAGTGGAGG
$KRAS^{K170R}$	GTCTTTTCTTCTTTGCTGATTCTTTTCAATCTGTATTGTCGGATCT	AGATCCGACAATACAGATTGAAAAGAATCAGCAAAGAAGAAAAGAC

KRAS kirsten rat sarcoma, $KRAS^{G12D}$ glycine to aspartic acid mutation of KRAS at G12 site, $KRAS^{K128R}$ lysine to arginine mutation of KRAS at K128 site, $KRAS^{K117R}$ lysine to arginine mutation of KRAS at K117 site, $KRAS^{K147R}$ lysine to arginine mutation of KRAS at K147 site, $KRAS^{K170R}$ lysine to arginine mutation of KRAS at K170 site

Table S3 Sequences of siRNAs

Gene (siRNA)	Sense (5' – 3')	Antisense (5' – 3')
si-NC	UUCUCCGAACGUGUCACGUTT	ACGUGACACGUUCGGAGAATT
si-Prelid2-1	GGACUCAUCUAUAGAAAGATT	UCUUUCUAUAGAUGAGUCCTT
si-Prelid2-2	GGACACAGUAUGCAUCCAUTT	AUGGAUGCAUACUGUGUCCTT
si-Prelid2-3	CUGGGUUUCUCAACUGUAUTT	AUACAGUUGAGAAACCCAGTT
si-Triap1-1	GCGUGCAGAAAGCAAUCAATT	UUGAUUGCUUUCUGCACGCTT
si-Triap1-2	AGUUCAUGGGCCAUGGCAATT	UUGCCAUGGCCCAUGAACUTT
si-Triap1-3	AUGAACAGCGUCGGGGAGGTT	CCUCCCCGACGCUGUUCAUTT

siRNA small interference RNAs, *Prelid2* proteins of relevant evolutionary and lymphoid interest (PRELI) domain 2, *Triap1* TP53 regulated inhibitor of apoptosis 1, *NC* negative control

Table S4 Primers for qPCR

Genes for mouse	Forward (5' – 3')	Reverse (5' – 3')
Afp	AGTTTCCAGAACCTGCCGAG	ACCTTGTCGTACTGAGCAGC
Gpc3	CGTTGGTGTAGTTCTTGGCA	CAACTAACAGCACGGCTGAA
Ly6d	CTCCACTGAGGTGACGGTTT	TCTGCTCGTCCTCCTTGTCT
Cd44	GTCCGGGAGATACTGTAGCG	CAAGTTTTGGTGGCACACAG
Ki67	AAAGGCGAAGTGGAGCTTCT	TTTCGCAACTTTCGTTTGTG
Pcna	AAAGATGCCGTCGGGTGAAT	CCATTGCCAAGCTCTCCACT
Ccnb1	AGCGAAGAGCTACAGGCAAG	CTCAGGCTCAGCAAGTTCCA
Ccnb2	CCGACGGTGTCCAGTGATTT	AGGTTTCTTCGCCACCTGAG
Icam1	CTGGGCTTGGAGACTCAGTG	CCACACTCTCCGGAAACGAA
Vcam1	CTGGGAAGCTGGAACGAAGT	GCCAAACACTTGACCGTGAC
Mmp9	CCTGGAACTCACACGACATCTTC	TGGAAACTCACACGCCAGAA
Ccl2	AAAAACCTGGATCGGAACCAA	CGGGTCAACTTCACATTCAAAG
Prelid2	CGAATTTCAATCACGGGGGC	GGGAGCCACACTGTTCCTTT
Slc41a3	CTGCCCTTCTCGCTTCCTC	TGTCCCTTCCATCAGCACAC
Gldn	CCAGCTTCAAAGGTAGGCCA	ATTGAGGCCAGAGCCAACTC
Triap1	ACCGCTGGTTTGCTGAGAAG	TCCTTGATTGCTTTCTGCACG
β-actin	TGTTACCAACTGGGACGACA	GGGGTGTTGAAGGTCTCAAA

qPCR quantitative PCR, *Afp* alpha fetoprotein, *Gpc3* glypican 3, *Ly6d* lymphocyte antigen 6 family member D, *Ki67* marker of proliferation Ki-67, *Pcna* proliferating cell nuclear antigen, *Ccnb1* cyclin B1, *Ccnb2* cyclin B2, *Icam1* intercellular adhesion molecule 1, *Vcam1* vascular cell adhesion molecule 1, *Mmp9* matrix metallopeptidase 9, *Ccl2* C-C motif chemokine ligand 2, *Prelid2* the proteins of relevant evolutionary and lymphoid interest (PRELI) domain 2, *Slc41a3* solute carrier family 41 member 3, *Gldn* gliomedin, *Triap1* TP53 regulated inhibitor of apoptosis 1

Table S5 Sequences of shRNAs

Gene (shRNAs)	Sequences (5' – 3')
sh-NC	TTCTCCGAACGTGTCACGT
sh-Prelid2-1	AGTCTGTCTTCCGGGAAAG
sh-Prelid2-2	AATGTGGTTCCAGAGATT
sh-Prelid2-3	GCTTGCTTCCTCCGAAAGT

shRNA short hairpin RNA, *NC* negative control, *Prelid2* the proteins of relevant evolutionary and lymphoid interest (PRELI) domain 2

Table S6 Relationships between PRELID2 and clinicopathologic characteristics in 129 HCC patients of the IHC cohort [*n*(%)]

**Variables**	**Total (*n* = 129)**	**PRELID2 low (*n* = 58)**	**PRELID2 high (*n* = 71)**	***P*-value**
Age (years)				0.091
<55	95(73.6)	38(65.5)	57(80.3)	
≥55	34(26.4)	20(34.5)	14(19.7)	
Gender				0.461
Female	20(15.5)	11(19.0)	9(12.7)	
Male	109(84.5)	47(81.0)	62(87.3)	
TNM stage				< 0.001
I – II	56(43.4)	35(60.3)	21(29.6)	
III – IV	73(56.6)	23(39.7)	50(70.4)	
Histologic grade				0.875
$G_1 – G_2$	106(82.2)	48(82.8)	58(81.7)	
G_3	23(17.8)	10(17.2)	13(18.3)	
Tumor size				0.013
≤ 5 cm	36(27.9)	23(39.7)	13(18.3)	
> 5 cm	93(72.1)	35(60.3)	58(81.7)	
Recurrence				< 0.001
Absent	39(30.2)	30(51.7)	9(12.7)	
Present	90(69.8)	28(48.3)	62(87.3)	
Vascular thrombosis				< 0.001
Absent	92(71.3)	51(87.9)	41(57.7)	
Present	37(28.7)	7(12.1)	30(42.3)	
Metastasis				< 0.001
Present	45(34.9)	11(19.0)	34(47.9)	
Absent	84(65.1)	47(81.0)	37(52.1)	

Statistical analyses were carried out using the Pearson χ^2 test. *PRELID2* the proteins of relevant evolutionary and lymphoid interest (PRELI) domain 2, *HCC* hepatocellular carcinoma, *IHC* immunohistochemistry

Table S7 Relationships between PRELID2 and clinicopathologic characteristics of HCC patients in the 365 HCC patient of TCGA database [*n*(%)]

**Characteristics**	**Total (*n* = 365)**	**PRELID2 low (*n* = 265)**	**PRELID2 high (*n* = 100)**	***P*-value**
Sex				0.271
Male	246(67.4)	183(69.1)	63(63.0)	
Female	119(32.6)	82(30.9)	37(37.0)	
Age (years)				0.743
≤ 60	173(47.4)	127(47.9)	46(46.0)	
> 60	192(52.6)	138(52.1)	54(54.0)	
UICC stage				< 0.001
I	170(46.6)	139(52.5)	31(31.0)	
II – IV	171(46.8)	112(42.3)	59(59.0)	
NA	24(6.6)	14(5.3)	10(10.0)	
Tumor stage				< 0.001
T_1	180(49.3)	146(55.1)	34(34.0)	
$T_2/T_3/T_4$	182(49.9)	116(43.4)	66(66.0)	
T_x	3(0.8)	3(1.1)	0	
Lymph node metastasis				0.416
Negative	248(67.9)	184(69.4)	64(64.0)	
Positive	4(1.1)	2(0.8)	2(2.0)	
Unknown	113(31.0)	79(29.8)	34(34.0)	
Cancer distant metastasis				0.557
Negative	263(72.1)	191(72.1)	72(72.0)	
Positive	3(0.8)	3(1.1)	0	
Unknown	99(27.1)	71(26.8)	28(28.0)	
Histologic grade				0.051
G_1	55(15.1)	39(14.7)	16(16.0)	
G_2	175(47.9)	138(52.1)	37(37.0)	
G_3	118(32.3)	77(29.1)	41(41.0)	

G_4	12(3.3)	9(3.4)	3(3.0)	
Gx	5(1.4)	2(0.8)	3(3.0)	
Vascular invasion				0.005
Negative	205(56.2)	159(60.0)	46(46.0)	
Positive	106(29.0)	76(28.7)	30(30.0)	
Data unavailable	54(14.8)	30(11.3)	24(24.0)	
Resection status				0.095
R_0	320(87.7)	237(89.4)	83(83.0)	
R_1	17(4.7)	8(3.0)	9(9.0)	
R_2	1(0.3)	1(0.4)	0	
Rx	27(7.4)	19(7.2)	8(8.0)	
Tumor states				0.019
With tumor	108(29.6)	77(29.1)	31(31.0)	
Tumor free	231(63.3)	175(66.0)	56(56.0)	
Data unavailable	26(7.1)	13(4.9)	13(13.0)	

Statistical analyses were carried out using the Pearson χ^2 test. *PRELID2* the proteins of relevant evolutionary and lymphoid interest (PRELI) domain 2, *HCC* hepatocellular carcinoma, *TCGA* The Cancer Genome Atlas

Table S8 Univariate and multivariate analyses indicating associations between overall survival and various risk factors in the 129 HCC patients of IHC cohort

**Variables**	***n***	**OS**	
		***HR* (95%CI)**	***P*-value**
Univariate analysis			
PRELID2 (high vs. low)	(71 vs. 58)	0.361(0.230 – 0.568)	<0.001
Age (≥ 55 years vs. < 55 years)	(34 vs. 95)	0.762(0.472 – 1.228)	0.264
Gender (male vs. female)	(109 vs. 20)	1.330(0.740 – 2.389)	0.340
Histologic grade (G_1 – G_2 vs. G_3)	(98 vs. 22)	0.768(0.459 – 1.285)	0.314
TNM stage (I – II vs. III – IV)	(56 vs. 73)	3.512(2.220 – 5.556)	<0.001
Tumor size (> 5 cm vs. ≤ 5 cm)	(93 vs. 36)	2.681(1.580 – 4.547)	<0.001
Recurrence (present vs. absent)	(90 vs. 39)	2.823(1.628 – 4.895)	<0.001
Vascular thrombosis (present vs. absent)	(37 vs. 92)	2.547(1.660 – 3.910)	<0.001
Metastasis (present vs. absent)	(45 vs. 84)	1.854(1.226 – 2.803)	0.003
Multivariate analysis			
PRELID2 (high vs. low)	(71 vs. 58)	0.546(0.325 – 0.915)	0.022
Tumor size (> 5 cm vs. ≤ 5 cm)	(93 vs. 36)	1.405(0.761 – 2.594)	0.278
Vascular thrombosis (present vs. absent)	(37 vs. 92)	1.033(0.616 – 1.730)	0.903
TNM stage (I – II vs. III – IV)	(56 vs. 73)	2.268(1.230 – 4.181)	0.009
Metastasis (present vs. absent)	(45 vs. 84)	0.959(0.612 – 1.504)	0.856
Recurrence (present vs. absent)	(90 vs. 39)	1.704(0.922 – 3.149)	0.089

PRELID2 the proteins of relevant evolutionary and lymphoid interest (PRELI) domain 2, *HCC* hepatocellular carcinoma, *IHC* immunohistochemistry

Table S9 The relationship between co-expression of FBXL6/p-ERK and clinicopathological features in 118 HCC patients of IHC cohort [*n*(%)]

**Variables**	**Total (*n* = 124)**	**FBXL6/p-ERK low (*n* = 51)**	**FBXL6/p-ERK high (*n* = 67)**	***P*-value**
Age (years)				0.705
< 55	87(73.7)	39(76.5)	48(71.6)	
⩾ 55	31(26.3)	12(23.5)	19(28.4)	
Gender				0.672
Female	20(16.9)	10(19.6)	10(14.9)	
Male	98(83.1)	41(80.4)	57(85.1)	
TNM stage				< 0.001
I – II	52(44.1)	35(68.6)	17(25.4)	
III – IV	66(55.9)	16(31.4)	50(74.6)	
Histologic grade				0.211
$G_1 – G_2$	97(82.2)	45(88.2)	52(77.6)	
G_3	21(17.8)	6(11.8)	15(22.4)	
Tumor size				0.029
⩽ 5 cm	35(29.7)	21(41.2)	14(20.9)	
> 5 cm	83(70.3)	30(58.8)	53(79.1)	
Recurrence				0.051
Absent	32(27.1)	19(37.3)	13(19.4)	
Present	86(72.9)	32(62.7)	54(80.6)	
Vascular thrombosis				< 0.001
Absent	85(72.0)	46(90.2)	39(58.2)	
Present	33(28.0)	5(9.8)	28(41.8)	
Metastasis				0.002
Present	43(36.4)	10(19.6)	33(49.3)	
Absent	75(63.6)	41(80.4)	34(50.7)	
Outcome				< 0.001

Dead	84(71.2)	26(51.0)	58(86.6)
Alive	34(28.8)	25(49.0)	9(13.4)

Statistical analyses were carried out using the Pearson χ^2 test. FBXL6 F-box and leucine-rich repeat 6, ERK extracellular signal-regulated kinase, IHC immunohistochemistry, HCC hepatocellular carcinoma

Table S10 Univariate and multivariate analyses indicating associations between overall survival and various risk factors in the 118 HCC patients of IHC cohort

**Variables**	***n***	**OS**	
		***HR* (95%CI)**	***P*-value**
Univariate analysis			
FBXL6 and p-ERK (high vs. low)	(67 vs. 51)	0.409(0.255 – 0.653)	< 0.001
Age (≥ 55 years vs. < 55 years)	(31 vs. 87)	0.692(0.415 – 1.154)	0.158
Gender (male vs. female)	(98 vs. 20)	1.225(0.678 – 2.214)	0.501
Histologic grade (G_1 – G_2 vs. G_3)	(97 vs. 21)	0.790(0.458 – 1.362)	0.396
TNM stage (I – II vs. III – IV)	(52 vs. 66)	3.506(2.161 – 5.688)	< 0.001
Tumor size (> 5 cm vs. ≤ 5 cm)	(83 vs. 35)	2.734(1.581 – 4.728)	< 0.001
Recurrence (present vs. absent)	(86 vs. 32)	4.431(2.262 – 8.682)	< 0.001
Vascular thrombosis (present vs. absent)	(33 vs. 85)	2.569(1.635 – 4.037)	< 0.001
Metastasis (present vs. absent)	(43 vs. 75)	2.054(1.330 – 3.170)	0.002
Multivariate analysis			
FBXL6 and p-ERK (high vs. low)	(67 vs. 51)	0.584(0.346 – 0.987)	0.044
TNM stage (I – II vs. III – IV)	(52 vs. 66)	2.041(1.076 – 3.870)	0.029
Tumor size (> 5 cm vs. ≤ 5 cm)	(83 vs. 35)	1.314(0.690 – 2.502)	0.407
Recurrence (present vs. absent)	(86 vs. 32)	3.436(1.672 – 7.059)	< 0.001
Vascular thrombosis (present vs. absent)	(33 vs. 85)	1.047(0.611 – 1.794)	0.867
Metastasis (present vs. absent)	(43 vs. 75)	0.954(0.591 – 1.538)	0.845

IHC immunohistochemistry, *HCC* hepatocellular carcinoma, *FBXL6* F-box and leucine-rich repeat 6, *ERK* extracellular signal-regulated kinase
